# Cave-adapted millipedes from Portugal: species conservation profiles

**DOI:** 10.3897/BDJ.11.e110382

**Published:** 2023-11-10

**Authors:** Ana Sofia P. S. Reboleira, Rita P. Eusébio

**Affiliations:** 1 Departamento de Biologia Animal and Centre for Ecology, Evolution and Environmental Changes (cE3c) & CHANGE - Global Change and Sustainability Institute, Faculdade de Ciências, Universidade de Lisboa, Lisbon, Portugal Departamento de Biologia Animal and Centre for Ecology, Evolution and Environmental Changes (cE3c) & CHANGE - Global Change and Sustainability Institute, Faculdade de Ciências, Universidade de Lisboa Lisbon Portugal; 2 Natural History Museum of Denmark, University of Copenhagen, Copenhagen, Denmark Natural History Museum of Denmark, University of Copenhagen Copenhagen Denmark

**Keywords:** Diplopoda, subterranean habitats, cave habitat, troglobiont, cave conservation, Iberian Peninsula

## Abstract

**Background:**

Amongst the cave-dwelling millipedes (Diplopoda), there are several endemic species in Portugal with a very small geographical distribution. These species play an important role in the decomposition of organic matter in subterranean ecosystems and are vulnerable to disturbance from human activities, such as habitat destruction, pollution infiltrating from the surface and cave tourism.

**New information:**

We present the IUCN Red List profiles for cave-adapted millipedes (Diplopoda) from Portugal and propose conservation measures to prevent extinction. Overall, cave-adapted millipedes from Portugal represent an endemic part of the country's biodiversity and conservation efforts will help maintain the delicate ecological balance of subterranean ecosystems.

## Introduction

Millipedes (Diplopoda) are key animals for biodiversity conservation, as they have limited dispersal capabilities and consequently exhibit high endemism patterns (Kime and Enghoff 2011, Kime and Enghoff 2017, Kime and Enghoff 2021). Millipedes contribute to organic matter decomposition, a fundamental biological process to ensure the ecological balance of terrestrial ecosystems, including in caves (Reboleira and Enghoff 2017, Ravn et al. 2020). Worldwide, millipedes have colonised the underground and exhibit unique convergent evolution of morphological adaptations that include elongation of the body, femora and tarsi of the walking legs, depigmentation and loss of eyes (Liu et al. 2017).

Portugal is a hotspot region for subterranean biodiversity and, despite a long tradition of studying different groups of subterranean arthropods, as crustaceans and beetles (Reboleira et al. 2011b, Reboleira et al. 2013, Reboleira and Eusébio 2021), the study of millipedes has been neglected until 2013, when the first cave-adapted millipede was described for science (Reboleira and Enghoff 2013). Over the last decade, our knowledge on cave millipede biodiversity and ecology increased significantly, expanding also towards the insular areas of the country with the description of the first cave-adapted millipedes from Madeira Island (Reboleira and Enghoff 2014b, Reboleira and Enghoff 2015, Reboleira and Enghoff 2016, Santamaria et al. 2017).

The recentlydescribed endemic millipede species face tremendous conservation challenges. Therefore, we created the IUCN Red List profile for cave millipedes from Portugal. This information aims to aid decisions about land-use at surface and territory planning and management.

## Material and Methods

During the last two decades, many millipedes from caves of Portugal have been sampled. All these specimens were studied and identified to species level, which included dissection, optical and scanning electronic microscopy study and comparison with other specimens and bibliography (Reboleira and Enghoff 2014a, Reboleira and Enghoff 2017, Reboleira and Enghoff 2018). This work was complemented with collections-based research and the study of museum specimens (Reboleira and Enghoff 2014b).

Both the extent of occurrence (EOO) and area of occupancy (AOO) were computed with the Geospatial Conservation Assessment Tool (GeoCAT) using an approximation to the IUCN standard 2 km × 2 km cells (4 km^2^). Software QGIS 3.14.16 was used to produce all maps, using the layer of natural protected areas of Portugal (ICNF 2023). The threats were evaluated *in situ*, through literature survey and spatial analysis, combining Google Earth satellite images. Based on the IUCN Red List database, we assign the type of habitat classification, threats and conservation actions; this included field visits and remote detection of threats, such as distance to quarries and urbanised areas.

Animals were photographed either alive *in situ* with a Cannon 6D mark II or preserved in alcohol with a stereomicroscope Leica DFC 420; images were processed with the Leica Application Suite, Zerene Stacker and background was cleaned in Adobe Photoshop CS5.

## Species Conservation Profiles

### Lusitanipus alternans

#### Species information

Scientific name: Lusitanipusalternans

Species authority: (Verhoeff, 1893)

Kingdom: Animalia

Phylum: Arthropoda

Class: Diplopoda

Order: Callipodida

Family: Dorypetalidae

Taxonomic notes:

Basionym: *Lysiopetalumalternans* Verhoeff, 1893.

*Lusitanipusalternans* and its congener *L.xanin* Gilgado, 2020 have similar gonopods, differing only in other variable characters. "*Lusitanipusxanin* sp. nov. differs from *L.alternans* in its green colour, the higher number of body rings, the shape of the gonocoxite and the curvature and shape of the processes of the tip of telopodites of gonopods" (in Gilgado et al. 2020). Therefore, the validity of *L.xanin* should be reconsidered in the future.

**Figure 1.**
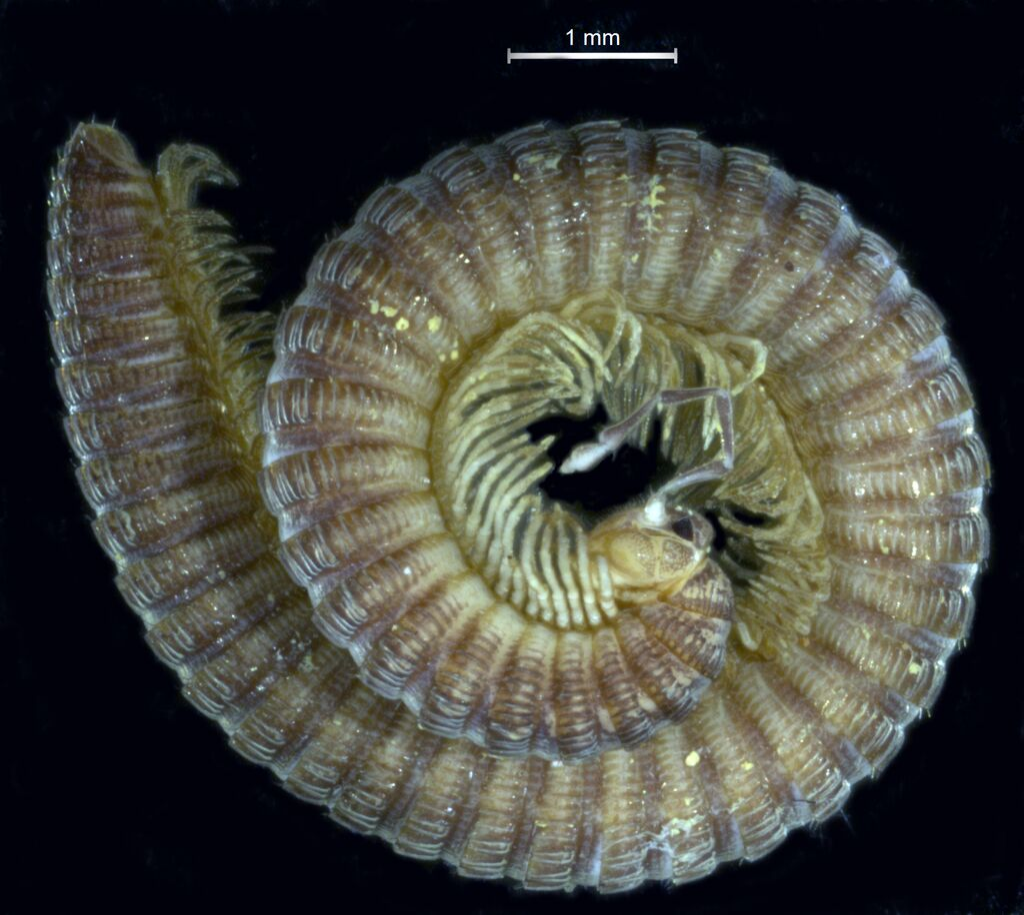
*Lusitanipusalternans* (Verhoeff, 1893), preserved female specimen from d’el Rey Cave in the Cantanhede-Outil karst massif. Picture taken from Reboleira & Enghoff 2015.

Figure(s) or Photo(s): Fig. 1

Region for assessment: Europe

#### Editor & Reviewers

#### Geographic range

Biogeographic realm: Palearctic

Countries: Portugal

Map of records (Google Earth): Suppl. materials 1, 2

Basis of EOO and AOO: Known habitat extent

Basis (narrative): The extent of occurrence (EOO) is 2470 km^2^ and area of occupancy (AOO) is 32 km^2^.

Range description: *Lusitanipusalternans* is recorded from eight localities: in d’el Rey, Soprador do Carvalho, Arrifana, Cerâmica, Corujeiras, Fonte Grande and Buraco da Moura caves and in the mesovoid shallow substratum from Poios Valley in Sicó karst massif (Reboleira and Enghoff 2015). The northernmost locality is Gruta d'El Rey in Portunhos, Cantanhede Municipality, Outil-Cantanhede karst massif. The easternmost locality is Buraco da Moura, in Lapa do Dinheiros, Serra da Estrela massif. All other localities are within Sicó-Condeixa and Alvaiázere karst area.

#### Extent of occurrence

EOO (km2): 2470

Trend: Decline (inferred)

Justification for trend: A decline in EOO is inferred because of the anthropogenic impact in the Soprador do Carvalho and Buraco da Moura caves. These caves show degradation signs, caused by the recreational visitation activities.

Causes ceased?: Unknown

Causes understood?: Unknown

Causes reversible?: Unknown

Extreme fluctuations?: Unknown

#### Area of occupancy

Trend: Decline (inferred)

Justification for trend: A decline in AOO is inferred due to the vulnerability of two of the caves.

Causes ceased?: Unknown

Causes understood?: Unknown

Causes reversible?: Unknown

Extreme fluctuations?: Unknown

AOO (km2): 32

#### Locations

Number of locations: 8

Justification for number of locations: *Lusitanipusalternans* occurs in eight locations in subterranean habitats, caves and mesovoid shallow substrate, in central Portugal (Reboleira and Enghoff 2015). It is recorded in Outil-Cantanhede massif, in caves and in the mesovoid shallow substrate thoughout the Sicó-Condeixa and Alvaiázere karst chains, down to Abrigo de Tomar I Cave in Ourém. It was recently found also in Serra da Estrela massif, in a cave formed by overlapping blocks of granite, the Buraco da Moura Cave.

Trend: Unknown

Extreme fluctuations?: Unknown

#### Population

Number of individuals: Unknown

Trend: Unknown

Causes ceased?: Unknown

Causes understood?: Unknown

Causes reversible?: Unknown

Extreme fluctuations?: Unknown

Population Information (Narrative): In caves, it is more abundant in d’el Rey Cave in Outil-Cantanhede karst area. It is also quite abundant in some caves of Sicó-Condeixa and Alvaiázere karst chains, such as Soprador do Carvalho, Arrifana, Corujeiras I and Fonte Grande caves, while in Cerâmica Cave, only juveniles have been collected (Reboleira and Enghoff 2015). In Sicó, it is also found in great numbers in the mesovoid shallow substratum (MSS) in scree slopes of Poio Valley. A disjunct population was recently found living in Buraco da Moura Cave in Lapa dos Dinheiros, Serra da Estrela massif.

#### Subpopulations

Number of subpopulations: 8

Trend: Unknown

Extreme fluctuations?: Unknown

Severe fragmentation?: Unknown

#### Habitat

System: Terrestrial

Habitat specialist: Yes

Habitat (narrative): *Lusitanipusalternans* is known from subterranean habitats. It lives in temperatures ranging from 12ºC in Buraco da Moura Cave up to 16.4ºC in Cerâmica Cave (Reboleira and Enghoff 2014a).

Trend in extent, area or quality?: Unknown

##### Habitat

Habitat importance: Major Importance

Habitats: 7.1. Caves and Subterranean Habitats (non-aquatic) - Caves

#### Ecology

Size: 35 mm (maximum body length), 2.25 mm (maximum body width).

Generation length (yr): 1

Dependency of single sp?: Unknown

Ecology and traits (narrative): *Lusitanipusalternans* is considered a troglophile species, i.e. a species whose presence is frequent in caves, but does not show specific adaptative morphologic traits to the subterranean lifestyle (Reboleira and Enghoff 2015). No information is available about old specimens collected or existence of type material (Reboleira and Enghoff 2015) and all efforts to retrieve surface speciments in their distribution area did not retrieve any specimens at the surface. Most specimens are highly infected with two species of ectoparasitic/ectobiont fungi of the order Laboulbeniales: *Diplopodomyceslusitanipodos* Santam., Enghoff & Reboleira, 2014 which can be mainly found on the legs, but also all along the body and *D.veneris* Santam., Enghoff & Reboleira, 2014, found exclusively on male gonopods and around female gonopores of *L.alternans* (Reboleira and Enghoff 2015). This species builds a moulting chamber composed of processed sediment reinforced with silk; it takes up to 35 days inside the moulting chamber before emerging and walking again in the cave floor (Reboleira and Enghoff 2016). These structures can be seen in the caves where the species occurs. The white secretions released by the ozopores from the defensive glands of *L.alternans* when disturbed, produce a very intense smell. The white secretions composed of p-cresol are characteristic for Callipodida and have antibacterial, antibiofilm and antifungal activity (Ilić et al. 2019). Therefore, their presence in caves is easily recognised by the typical odour in the cave's substrate.

#### Threats

Justification for threats: D‘el Rey Cave is located in an urbanised area, 600 m from a landfill, 1 km from a quarry and 1.2 km from highway A14. Arrifana Cave is located 190 m from a road, 370 m from the nearest village and 900 m from a quarry. Cerâmica Cave is surrounded by *Eucalyptus* intensive plantations and is located 270 m from a road, 550 m from an animal farm, 1.6 km from the closest village and 3.6 km from a quarry. Soprador do Carvalho Cave is surrounded by agricultural lands and is located 67 m from the closest house and 1.4 km from a quarry. This is a touristic cave, severely affected by anthropogenic activities (Ribera and Reboleira 2019). The subterranean stream that flows inside the Soprador do Carvalho Cave has urban wastewater run-off (Reboleira et al. 2011b). Corujeiras Cave is located 63 m from the closest house, 500 m from the warehouse complex of a transportation and shipping company and 700 m from the closest village. Fonte Grande Cave is located in an intensive agricultural area, 240 m away from the closest house, 500 m from a metalwork construction company and right beside the main road from where the construction material is transported, located 480 m from the closest village. Buraco da Moura Cave is located 127 m from a fluvial beach, 530 m from a hydroelectric power station and 1.2 km from the closest village and is under anthropogenic disturbance due to frequent touristic visits (Reboleira and Eusébio 2021).

##### Threats

Threat type: Ongoing

Threats: 1.1. Residential & commercial development - Housing & urban areas2.2. Agriculture & aquaculture - Wood & pulp plantations2.3. Agriculture & aquaculture - Livestock farming & ranching3.2. Energy production & mining - Mining & quarrying4.1. Transportation & service corridors - Roads & railroads6.1. Human intrusions & disturbance - Recreational activities9.1. Pollution - Domestic & urban waste water9.3. Pollution - Agricultural & forestry effluents9.4. Pollution - Garbage & solid waste

#### Conservation

Justification for conservation actions: Of the eight locations, only three are inside the “Rede Natura 2000” areas (Directive 1992, ICNB 2000). Measures should be taken to prevent infiltration of wastewaters from villages and quarries into the subterranean ecosystems and to minimise the effects of anthropogenic threats on the habitats and consequently for this species. The mesovoid shallow substratum where the species occour, i.e. in Sicó massif, should be also protected, to ensure the stabilisation of the populations. Molecular approaches are needed to understand populations structure and degree of isolation in the subterranean ecosystem.

##### Conservation actions

Conservation action type: Needed

Conservation actions: 1.1. Land/water protection - Site/area protection2.1. Land/water management - Site/area management2.3. Land/water management - Habitat & natural process restoration5.1.3. Law & policy - Legislation - Sub-national level4.1. Education & awareness - Formal education4.3. Education & awareness - Awareness & communications

### Sireuma nobile

#### Species information

Scientific name: Sireumanobile

Species authority: Reboleira & Enghoff, 2014

Kingdom: Animalia

Phylum: Arthropoda

Class: Diplopoda

Order: Chordeumatida

Family: Opisthocheiridae

Taxonomic notes: This species is included in a monospecific genus and is distinguishable from other opisthocheirids due to the “anterior male gonopods with a distinct small anterior synangiocoxite and separate, two-segmented colpocoxites enfolded by biramous, very large telepodites; posterior gonopods two-segmented, without processes; first pair of postgonopodal legs (P10) with a set of three strong setae next to coxal gland openings; vulvae without a postvulvar organ” (Reboleira and Enghoff 2014c).

Region for assessment: Europe

#### Editor & Reviewers

#### Geographic range

Biogeographic realm: Palearctic

Countries: Portugal

Map of records (Google Earth): Suppl. materials 1, 3

Basis of EOO and AOO: Known habitat extent

Basis (narrative): Both the extent of occurrence (EOO) and area of occupancy (AOO) are 4 km^2^.

Max Elevation/Depth (m): 350

Range description: *Sireumanobile* is known only from a single cave, the Algar de Santo António in the Estremoz-Cano karst massif (Reboleira and Enghoff 2014c).

#### Extent of occurrence

EOO (km2): 4

Trend: Unknown

Causes ceased?: Unknown

Causes understood?: Unknown

Causes reversible?: Unknown

Extreme fluctuations?: Unknown

#### Area of occupancy

Trend: Unknown

Causes ceased?: Unknown

Causes understood?: Unknown

Causes reversible?: Unknown

Extreme fluctuations?: Unknown

AOO (km2): 4

#### Locations

Number of locations: 1

Justification for number of locations: *Sireumanobile* occurs in a single cave (Reboleira and Enghoff 2014c).

Trend: Stable

Justification for trend: Algar de Santo António is the only known location for this species. Therefore, the trend in number of locations is stable.

Extreme fluctuations?: Unknown

#### Population

Number of individuals: Unknown

Trend: Unknown

Causes ceased?: Unknown

Causes understood?: Unknown

Causes reversible?: Unknown

Extreme fluctuations?: Unknown

Population Information (Narrative): Fifteen specimens have been collected from the type location (Reboleira and Enghoff 2014c).

#### Subpopulations

Number of subpopulations: 1

Trend: Unknown

Extreme fluctuations?: Unknown

Severe fragmentation?: Unknown

#### Habitat

System: Terrestrial

Habitat specialist: Yes

Habitat (narrative): Algar de Santo António Cave is composed of two entrances that connect at the base of the first pit and it has a a maximum depth of -52 m, currently ending in rubble blocks. *Sireumanobile* specimens were collected at -20 and -52 m. Inside the cave, humidity levels reach 100% and temperature ranges from 17.7ºC up to 18.9ºC at the soil level (Reboleira and Enghoff 2014c).

Trend in extent, area or quality?: Unknown

##### Habitat

Habitat importance: Major Importance

Habitats: 7.1. Caves and Subterranean Habitats (non-aquatic) - Caves

#### Ecology

Size: 5–6 mm (body length), 0.5–0.6 mm (vertical body diameter).

Generation length (yr): 1

Dependency of single sp?: Unknown

Ecology and traits (narrative): *Sireumanobile* is a troglobiont species, depigmented and anophthalmic (Reboleira and Enghoff 2014c). It shares habitat with other subterranean detritivores, like the terrestrial isopods *Trichorhinaanophthalma* Arcangeli, 1936 and *Cordioniscuslusitanicus* Reboleira & Taiti, 2015, several unidentified species of Collembola, new species of Zygentoma from the genus *Coletinia*, new Anillini beetle of the genus *Geocharis* and histerid beetles (Reboleira and Enghoff 2014c).

#### Threats

Justification for threats: Algar de Santo António Cave is located in the middle of an urbanised area of a village, under a building and right by a road. It has a building on top of the main entrance and its distance is 27 m from the nearest house. The location is surrounded by agricultural fields. There is a massive quarry located 3 km from the cave entrance, where stone is extracted and prepared in slabs for retail.

##### Threats

Threat type: Ongoing

Threats: 1.1. Residential & commercial development - Housing & urban areas2.2. Agriculture & aquaculture - Wood & pulp plantations3.2. Energy production & mining - Mining & quarrying4.1. Transportation & service corridors - Roads & railroads9.1. Pollution - Domestic & urban waste water

#### Conservation

Justification for conservation actions: This cave is located out of the “Rede Natura 2000” areas (Directive 1992, ICNB 2000), within the village of Alandroal. The cave should be protected in order to prevent extinction of this single cave endemic. It is essential to carry out new research to better understand its potential distribution, ecology and evolution, which support the preparation of a conservation plan for the species. In addition to the regular monitoring of its population, it is also recommended to prospect other areas of potential occurrence, including the deep soil layers around the cave. There should be a concerted effort by the competent authorities to mitigate the negative impacts of threats in the species' occurrence zones by limiting anthropogenic disturbance.

##### Conservation actions

Conservation action type: Needed

Conservation actions: 1.1. Land/water protection - Site/area protection2.1. Land/water management - Site/area management2.3. Land/water management - Habitat & natural process restoration4. Education & awareness5.1.3. Law & policy - Legislation - Sub-national level

### Scutogona minor

#### Species information

Scientific name: Scutogonaminor

Species authority: Enghoff & Reboleira, 2013

Kingdom: Animalia

Phylum: Arthropoda

Class: Diplopoda

Order: Chordeumatida

Family: Chamaesomatidae

Taxonomic notes: *Scutogonaminor* adults differ from other species in the same genus due to having 29 pleurotergites, a densely pilose head, a tridentate labrum and strongly protruding subglobular mandibular stipites (Enghoff and Reboleira 2013a).

Region for assessment: Europe

#### Editor & Reviewers

#### Geographic range

Biogeographic realm: Palearctic

Countries: Portugal

Map of records (Google Earth): Suppl. materials 1, 4

Basis of EOO and AOO: Known habitat extent

Basis (narrative): The extent of occurrence (EOO) is 25896 km^2^ and the area of occupancy (AOO) is 12 km^2^.

Range description: *Scutogonaminor* is known from three caves located in the Sicó karst massif, in central Portugal: Santa Maria da Estrela, Cerâmica and Arrifana caves (Enghoff and Reboleira 2013a).

#### Extent of occurrence

EOO (km2): 25896

Trend: Unknown

Causes ceased?: Unknown

Causes understood?: Unknown

Causes reversible?: Unknown

Extreme fluctuations?: Unknown

#### Area of occupancy

Trend: Unknown

Causes ceased?: Unknown

Causes understood?: Unknown

Causes reversible?: Unknown

Extreme fluctuations?: Unknown

AOO (km2): 12

#### Locations

Number of locations: 3

Justification for number of locations: *Scutogonaminor* occurs in three locations, three caves within the Sicó karst area (Enghoff and Reboleira 2013a).

Trend: Unknown

Extreme fluctuations?: Unknown

#### Population

Number of individuals: Unknown

Trend: Unknown

Causes ceased?: Unknown

Causes understood?: Unknown

Causes reversible?: Unknown

Extreme fluctuations?: Unknown

Population Information (Narrative): It was found in higher numbers in Arrifana Cave, in the winter season, followed by Santa Maria da Estrela Cave (Enghoff and Reboleira 2013a) and by Cerâmica Cave, where several specimens have been sampled more recently.

#### Subpopulations

Number of subpopulations: 3

Trend: Unknown

Extreme fluctuations?: Unknown

Severe fragmentation?: Unknown

#### Habitat

System: Terrestrial

Habitat specialist: Yes

Habitat (narrative): The maximum distance of the known distribution is of 18 km between the three caves. Specimens were only collected in the deepest parts of the caves and they were collected live through active search. All caves have high humidity levels (up to 100%) and at soil level, temperature ranged from 15.3ºC to 16.4ºC (Enghoff and Reboleira 2013a).

Trend in extent, area or quality?: Unknown

##### Habitat

Habitat importance: Major Importance

Habitats: 7.1. Caves and Subterranean Habitats (non-aquatic) - Caves

#### Ecology

Size: 5–6 mm (body length), 0.45–0.5 mm (vertical body diameter).

Generation length (yr): 1

Dependency of single sp?: Unknown

Ecology and traits (narrative): *Scutogonaminor* is the smallest species of its genus. It is a troglobiont species, depigmented, lacks eyes and is endemic from caves in the Sicó massif (Enghoff and Reboleira 2013a). It shares habitat with several cave-adapted species, such as the terrestrial isopods *Trichoniscoidessicoensis* Reboleira & Taiti, 2015, *Miktoniscuslongispina* Reboleira & Taiti, 2015 and *Porcelliocavernicolus* Vandel, 1946 (Reboleira et al. 2015, Reboleira et al. 2022) and the dipluran Podocampacf.fragiloides Silvestri, 1932 (Sendra and Reboleira 2020). These caves are also inhabited by cave-adapted predators, the pseudoscorpions *Roncocreagrisblothroides* (Beier, 1962), *R.borgesi* Zaragoza & Reboleira, 2013 (Reboleira et al. 2013) and *Occidenchthoniusvachoni* Zaragoza & Reboleira, 2018 (Zaragoza and Reboleira 2018) and the rove beetle *Domenelusitanica* Reboleira & Oromí, 2011 (Reboleira et al. 2011a, Reboleira and Eusébio 2021).

#### Threats

Justification for threats: Arrifana Cave is located 190 m from a road, 370 m from the nearest village and 900 m from a quarry. Cerâmica Cave is surrounded by *Eucalyptus* intensive plantations and is located 270 m from a road, 550 m from an animal farm, 1.6 km from the closest village and 3.6 km from a quarry. Santa Maria da Estrela Cave is located 80 m from a road, 86 m from a touristic site, 220 m from agricultural fields, 230 m from the Nossa Senhora da Estrela viewpoint, 250 m from the closest urbanised area and 2.6 km from two quarries. All caves have geocaches inside, which means that their entrances are frequently visited by geocachers.

##### Threats

Threat type: Ongoing

Threats: 1.1. Residential & commercial development - Housing & urban areas2.1. Agriculture & aquaculture - Annual & perennial non-timber crops2.3. Agriculture & aquaculture - Livestock farming & ranching3.2. Energy production & mining - Mining & quarrying4. Transportation & service corridors6.1. Human intrusions & disturbance - Recreational activities9.1. Pollution - Domestic & urban waste water

#### Conservation

Justification for conservation actions: This species has a very reduced distribution, confined to three caves in the Sicó Karst Massif and face several threats. It is important to define an undisturbed surface area to ensure that no contaminants, such as pesticides from agriculture and effluents from farms do not infiltrate and impact these populations. Geocaches should be removed from the caves to prevent their use by tourists. Biological prospection in other caves within the area of distribution have the potential to expand the currently-known populations of *Scutogonaminor*.

##### Conservation actions

Conservation action type: Needed

Conservation actions: 1.1. Land/water protection - Site/area protection2.1. Land/water management - Site/area management2.3. Land/water management - Habitat & natural process restoration4. Education & awareness5.1.3. Law & policy - Legislation - Sub-national level

### Boreviulisoma barrocalense

#### Species information

Scientific name: Boreviulisomabarrocalense

Species authority: Reboleira & Enghoff, 2013

Kingdom: Animalia

Phylum: Arthropoda

Class: Diplopoda

Order: Polydesmida

Family: Paradoxosomatidae

Taxonomic notes: Distinguishable from other *Boreviulisoma* species by being depigmented, by “having femoral knobs on male legs 3–7 and 9–10” and by “having a hawksbill-like process on the dorsal side of the gonopod tip” (Reboleira and Enghoff 2013).

**Figure 2.**
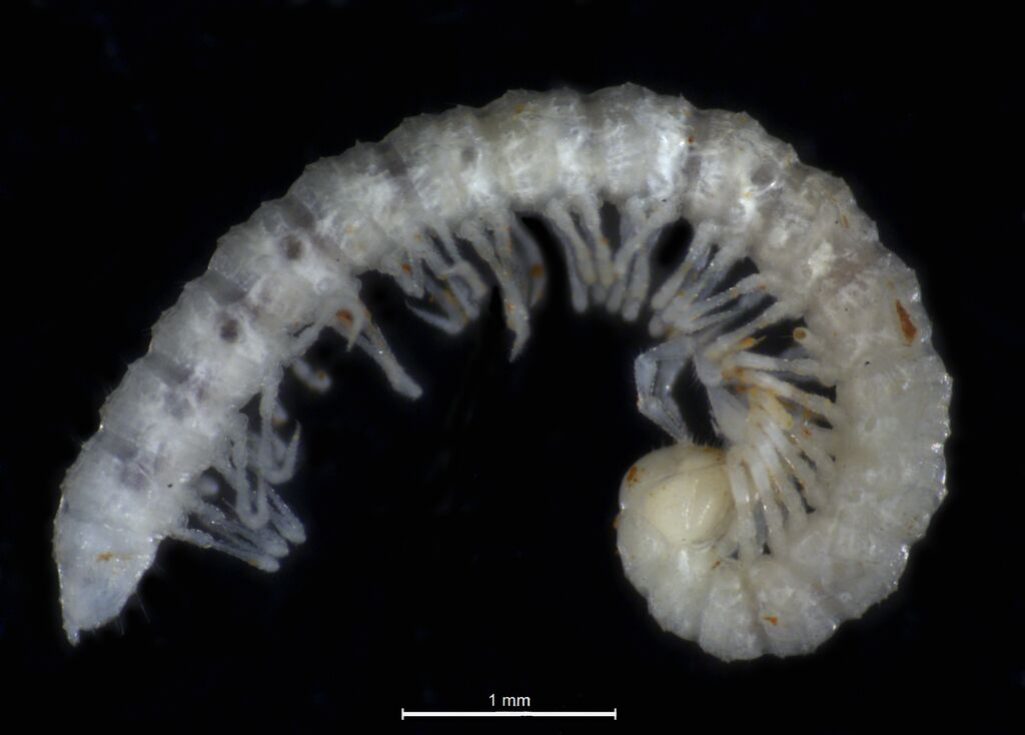
*Boreviulisomabarrocalense* Reboleira & Enghoff, 2013, preserved female specimen from Vale Telheiro Cave in the Algarve karst massif.

Figure(s) or Photo(s): Fig. 2

Region for assessment: Europe

#### Editor & Reviewers

#### Geographic range

Biogeographic realm: Palearctic

Countries: Portugal

Map of records (Google Earth): Suppl. materials 1, 5

Basis of EOO and AOO: Known habitat extent

Basis (narrative): Both the extent of occurrence (EOO) and area of occupancy (AOO) are 4 km^2^.

Max Elevation/Depth (m): 239

Range description: *Boreviulisomabarrocalense* occurs in Vale Telheiro Cave, in the Algarve karst massif, southern Portugal (Reboleira and Enghoff 2013).

#### Extent of occurrence

EOO (km2): 4

Trend: Unknown

Causes ceased?: Unknown

Causes understood?: Unknown

Causes reversible?: Unknown

Extreme fluctuations?: Unknown

#### Area of occupancy

Trend: Unknown

Causes ceased?: Unknown

Causes understood?: Unknown

Causes reversible?: Unknown

Extreme fluctuations?: Unknown

AOO (km2): 4

#### Locations

Number of locations: 1

Justification for number of locations: *Boreviulisomabarrocalense* occurs in one location (Reboleira and Enghoff 2013).

Trend: Stable

Justification for trend: Vale Telheiro Cave is the only known location for this species. Therefore, the trend in number of locations is stable.

Extreme fluctuations?: Unknown

#### Population

Number of individuals: Unknown

Trend: Unknown

Causes ceased?: Unknown

Causes understood?: Unknown

Causes reversible?: Unknown

Extreme fluctuations?: Unknown

Population Information (Narrative): Five specimens have been collected from the type locality (Reboleira and Enghoff 2013).

#### Subpopulations

Trend: Unknown

Extreme fluctuations?: Unknown

Severe fragmentation?: Unknown

#### Habitat

System: Terrestrial

Habitat specialist: Yes

Habitat (narrative): Vale Telheiro Cave, is currently the most biodiverse cave in terms of troglobiont species in Portugal (Reboleira & Enghoff 2013), accounting now for more than 25 species, which turns it into a world hotspot for subterranean biodiversity. It has high humidity levels and very stable environment conditions (Reboleira et al. 2017), as well as reduced levels of oxygen in its deeper parts.

Trend in extent, area or quality?: Unknown

##### Habitat

Habitat importance: Major Importance

Habitats: 7.1. Caves and Subterranean Habitats (non-aquatic) - Caves

#### Ecology

Size: 9–10 mm (♂) and 10–11 mm (♀) (body length).

Generation length (yr): 1

Dependency of single sp?: Unknown

Ecology and traits (narrative): *Boreviulisomabarrocalense* is a troglobiont species (Reboleira and Enghoff 2013), endemic to the Vale Telheiro Cave. This cave is located 240 m a.s.l. in Algarve, the southernmost province of Portugal and has a very stable temperature, ranging from 17.1 up to 17.4°C (during the entire 2020). This species shares habitat with other several cave-adapted species, such as the pseudoscorpions *Occidenchthoniusalgharbicus* Zaragoza & Reboleira, 2018 and *Titanobochicamagna* Zaragoza & Reboleira, 2020 ([Bibr B7887202][Bibr B7884009]), the spiders *Harpacteastalitoides* Ribera, 1993 and *Teloleptonetasynthetica* (Machado, 1951) (Reboleira 2012), the millipede *Acipesmachadoi* Enghoff & Reboleira, 2013 (Enghoff and Reboleira 2013b), the terrestrial isopods *Cordioniscuslusitanicus* Reboleira & Taiti, 2015 and *Troglelumamachadoi* (Vandel, 1946) (Reboleira et al. 2015, Reboleira et al. 2022), the dipluran *Litocampamendesi* Sendra & Reboleira, 2010 (Reboleira et al. 2010a, Sendra and Reboleira 2020), the zygentoma *Squamatiniaalgharbica* Mendes & Reboleira, 2012 (Reboleira et al. 2012) and the beetle *Speonemadusalgarvensis* Reboleira, Fresneda & Salgado, 2017 (Reboleira et al. 2017, Reboleira and Eusébio 2021).

#### Threats

Justification for threats: Vale Telheiro is located 290 m from the closest house and 745 m from the closest urbanisation. The immediate surface of the cave has recently been subject to landfills and the closest road has been enlarged and tarred, facilitating the access to the cave area.

##### Threats

Threat type: Ongoing

Threats: 1.1. Residential & commercial development - Housing & urban areas4. Transportation & service corridors9.1. Pollution - Domestic & urban waste water

#### Conservation

Justification for conservation actions: Vale Telheiro Cave, where these single cave endemics occur must be protected. As the terrain where the cave is located was acquired by the Loulé Town Hall in order to establish a protected area, it is possible to foresee more conservation efforts in the near future. The surface area should be maintained with its natural vegetation cover and cave visitation should be limited. Efforts should be made to understand the spacio-temporal dynamics and life cycle of this species, also the population trends should to be monitored. This species, together with the cave-adapted millipede *Acipesmachadoi*, inhabits the richest cave for troglobiont species in Portugal and protecting this species implies the protection this important habitat.

##### Conservation actions

Conservation action type: Needed

Conservation actions: 1.1. Land/water protection - Site/area protection2.1. Land/water management - Site/area management2.3. Land/water management - Habitat & natural process restoration4. Education & awareness5.1.3. Law & policy - Legislation - Sub-national level

### Cylindroiulus julesvernei

#### Species information

Scientific name: Cylindroiulusjulesvernei

Species authority: Reboleira & Enghoff, 2014

Kingdom: Animalia

Phylum: Animalia

Class: Diplopoda

Order: Julida

Family: Julidae

Taxonomic notes: Distinguishable from all other species of the *Cylindroiulusmadeirae*-group by the hook-shaped and higher than promerite gonopod mesomerite (Reboleira and Enghoff 2014b) and from all, except Cylindroiulus *oromii* Reboleira & Enghoff, 2014, by its depigmented body and loss of eyes.

Region for assessment: Europe

#### Editor & Reviewers

#### Geographic range

Biogeographic realm: Palearctic

Countries: Portugal

Map of records (Google Earth): Suppl. materials 1, 6

Basis of EOO and AOO: Known habitat extent

Basis (narrative): Both the extent of occurrence (EOO) and area of occupancy (AOO) are 4 km^2^.

Range description: *Cylindroiulusjulesvernei* is endemic from São Vicente Cave, in the Madeira Archipelago (Reboleira and Enghoff 2014b).

#### Extent of occurrence

EOO (km2): 4

Trend: Decline (inferred)

Justification for trend: A decline in EOO is inferred due to the anthropogenic impact on the cave, as the visitors directly trample on the cave substrate.

Causes ceased?: Unknown

Causes understood?: Unknown

Causes reversible?: Unknown

Extreme fluctuations?: Unknown

#### Area of occupancy

Trend: Decline (inferred)

Justification for trend: The decline of AOO is inferred due to current threats to the habitat.

Causes ceased?: Unknown

Causes understood?: Unknown

Causes reversible?: Unknown

Extreme fluctuations?: Unknown

AOO (km2): 4

#### Locations

Number of locations: 1

Justification for number of locations: *Cylindroiulusjulesvernei* occurs in one location (Reboleira and Enghoff 2014b).

Trend: Stable

Justification for trend: São Vicente Cave is the only known location for this species. Therefore, the trend in number of locations is stable.

Extreme fluctuations?: Unknown

#### Population

Number of individuals: Unknown

Trend: Unknown

Causes ceased?: Unknown

Causes understood?: Unknown

Causes reversible?: Unknown

Extreme fluctuations?: Unknown

Population Information (Narrative): All three known specimens have been collected from the type locality (Reboleira and Enghoff 2014b).

#### Subpopulations

Trend: Unknown

Extreme fluctuations?: Unknown

Severe fragmentation?: Unknown

#### Habitat

System: Terrestrial

Habitat specialist: Yes

Habitat (narrative): São Vicente Cave is a lava tube, formed 890 thousand years ago after a volcanic eruption at Paul da Serra (Reboleira and Enghoff 2014b).

Trend in extent, area or quality?: Unknown

##### Habitat

Habitat importance: Major Importance

Habitats: 7.1. Caves and Subterranean Habitats (non-aquatic) - Caves

#### Ecology

Size: 1.0 mm (vertical body diameter).

Generation length (yr): 1

Dependency of single sp?: Unknown

Ecology and traits (narrative): This species is a troglobiont species, blind and depigmented (Reboleira and Enghoff 2014b).

#### Threats

Justification for threats: This is a show cave, opened since 1996 to the public and can be visited by tourists through a route of 700 m. The cave has artificial light and, as a consequence, lampenflora proliferate around the light sources. This cave is located at the centre of an urbanised area, close to roads.

##### Threats

Threat type: Ongoing

Threats: 1.1. Residential & commercial development - Housing & urban areas4. Transportation & service corridors6.1. Human intrusions & disturbance - Recreational activities9.1. Pollution - Domestic & urban waste water

#### Conservation

Justification for conservation actions: This cave is a show cave and is not protected under legislation by the “Rede Natura 2000” (Directive 1992, ICNB 2000). Seeing that this species is an island single cave endemic, its protection is of grave importance.

##### Conservation actions

Conservation action type: Needed

Conservation actions: 1.1. Land/water protection - Site/area protection2.1. Land/water management - Site/area management2.3. Land/water management - Habitat & natural process restoration4. Education & awareness5.1.3. Law & policy - Legislation - Sub-national level

### Cylindroiulus oromii

#### Species information

Scientific name: Cylindroiulusoromii

Species authority: Reboleira & Enghoff, 2014

Kingdom: Animalia

Phylum: Arthropoda

Class: Diplopoda

Order: Julida

Family: Julidae

Taxonomic notes: Distinguished from all species of the *Cylindroiulusmadeirae*-group, except *Cylindroiulusjulesvernei* Reboleira & Enghoff, 2014, by depigmented body and lack of eyes. It is also distinguished from *C.julesvernei* by having the mesomere not hooked (male gonopod) (Reboleira and Enghoff 2014b).

**Figure 3.**
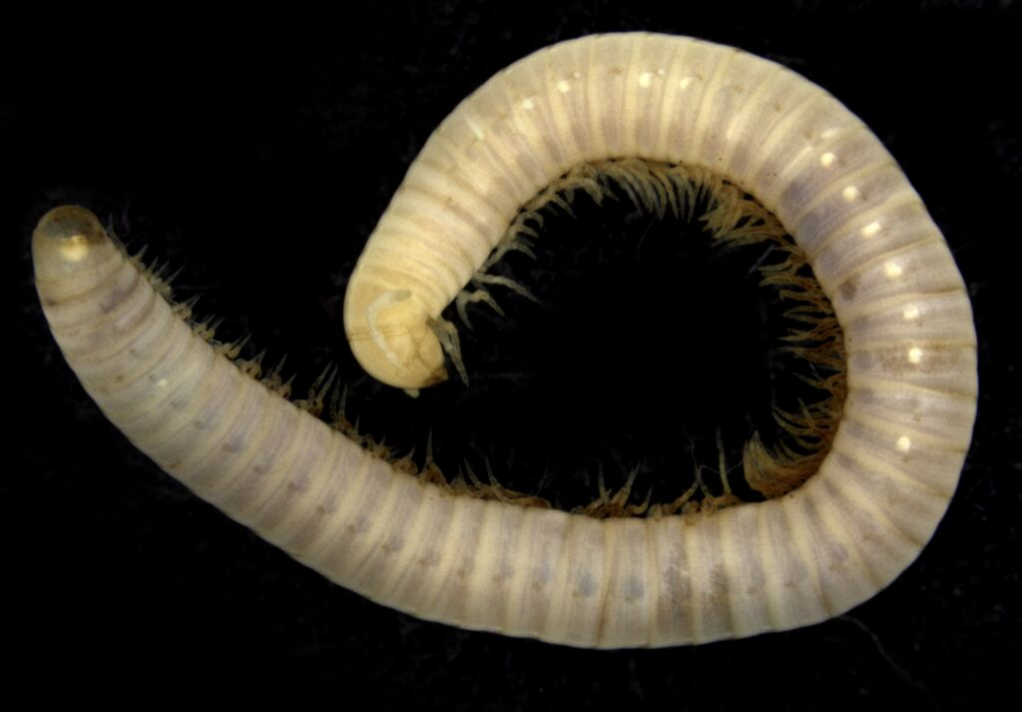
*Cylindroiulusoromii* Reboleira & Enghoff, 2014, preserved female specimen from Landeiros Cave, Madeira, Portugal.

Figure(s) or Photo(s): Fig. 3

Region for assessment: Europe

#### Editor & Reviewers

#### Geographic range

Biogeographic realm: Palearctic

Countries: Portugal

Map of records (Google Earth): Suppl. materials 1, 7

Basis of EOO and AOO: Known habitat extent

Basis (narrative): Both the extent of occurrence (EOO) and area of occupancy (AOO) are approximately 4 km^2^.

Max Elevation/Depth (m): 200

Range description: *Cylindroiulusoromii* occurs in Landeiros Cave, in the Madeira Archipelago (Reboleira and Enghoff 2014b).

#### Extent of occurrence

EOO (km2): 4

Trend: Unknown

Causes ceased?: Unknown

Causes understood?: Unknown

Causes reversible?: Unknown

Extreme fluctuations?: Unknown

#### Area of occupancy

Trend: Unknown

Causes ceased?: Unknown

Causes understood?: Unknown

Causes reversible?: Unknown

Extreme fluctuations?: Unknown

AOO (km2): 4

#### Locations

Number of locations: 1

Justification for number of locations: *Cylindroiulusoromii* occurs in one location (Reboleira and Enghoff 2014b).

Trend: Stable

Justification for trend: Landeiros Cave is the only known location for this species. Therefore, the trend in number of locations is stable.

Extreme fluctuations?: Unknown

#### Population

Number of individuals: Unknown

Trend: Unknown

Causes ceased?: Unknown

Causes understood?: No

Causes reversible?: Unknown

Extreme fluctuations?: Unknown

Population Information (Narrative): All known specimens have been collected from the type locality (Reboleira and Enghoff 2014b).

#### Subpopulations

Trend: Unknown

Extreme fluctuations?: Unknown

Severe fragmentation?: Unknown

#### Habitat

System: Terrestrial

Habitat specialist: Yes

Habitat (narrative): Landeiros Cave is a lava tube, located within the volcanic complex of São Roque/Paúl (SRP), in the Santo da Serra lava flow. The mean temperature inside the cave is 16ºC to 17ºC (Reboleira and Enghoff 2014b).

Trend in extent, area or quality?: Unknown

##### Habitat

Habitat importance: Major Importance

Habitats: 7.1. Caves and Subterranean Habitats (non-aquatic) - Caves

#### Ecology

Size: 1.1 mm (♂), 1.34 mm (♀) (vertical body diameter).

Generation length (yr): 1

Dependency of single sp?: Unknown

Ecology and traits (narrative): *C.oromii* is bilnd and depigmented and shares habitat with the troglobiont carabid beetle *Thalassophiluspieperi* Erber, 1990 and several troglophile species, like the *snail Oxychilus draparnaudi* (Beck, 1837), the mites *Veigaiauncata* Farrier, 1957 and *Uroseiusacuminatus* (Koch, 1847), the spider *Steatodagrossa* (Koch, 1838), the terrestrial isopod *Soteriscus* sp., the centipede *Lithobius* sp., two species of springtails from the genus *Onychiurus* Gervais, 1841, the carabid beetle *Trechusfulvus* Dejean, 1831 and several Phoridae and Psychodidae dipterans (Reboleira and Enghoff 2014b).

#### Threats

Justification for threats: Landeiros Cave is located below agricultural terrains and right by urbanised areas and roads, in the island of Madeira.

##### Threats

Threat type: Ongoing

Threats: 1.1. Residential & commercial development - Housing & urban areas4. Transportation & service corridors

#### Conservation

Justification for conservation actions: The only known locality for this species is a non-protected cave; therefore, *C.oromii* is a single cave endemic living in a Darwinian island. The cave and its respective surface should be priority targets for conservation, in order to prevent infiltration from pesticides and insecticides used in agriculture. More research is needed to understand the life cycle of this species.

##### Conservation actions

Conservation action type: Needed

Conservation actions: 1.1. Land/water protection - Site/area protection2.1. Land/water management - Site/area management2.3. Land/water management - Habitat & natural process restoration4. Education & awareness5.1.3. Law & policy - Legislation - Sub-national level

### Cylindroiulus villumi

#### Species information

Scientific name: Cylindroiulusvillumi

Species authority: Reboleira & Enghoff, 2018

Kingdom: Animalia

Phylum: Arthropoda

Class: Diplopoda

Order: Julida

Family: Julidae

Taxonomic notes: Distinguishable from other species of the *Cylindroiulusperforatus*-group by being blind and by “the shape of the gonopod mesomerite, which is shorter than the promerite and apically rounded” (Reboleira and Enghoff 2018).

**Figure 4.**
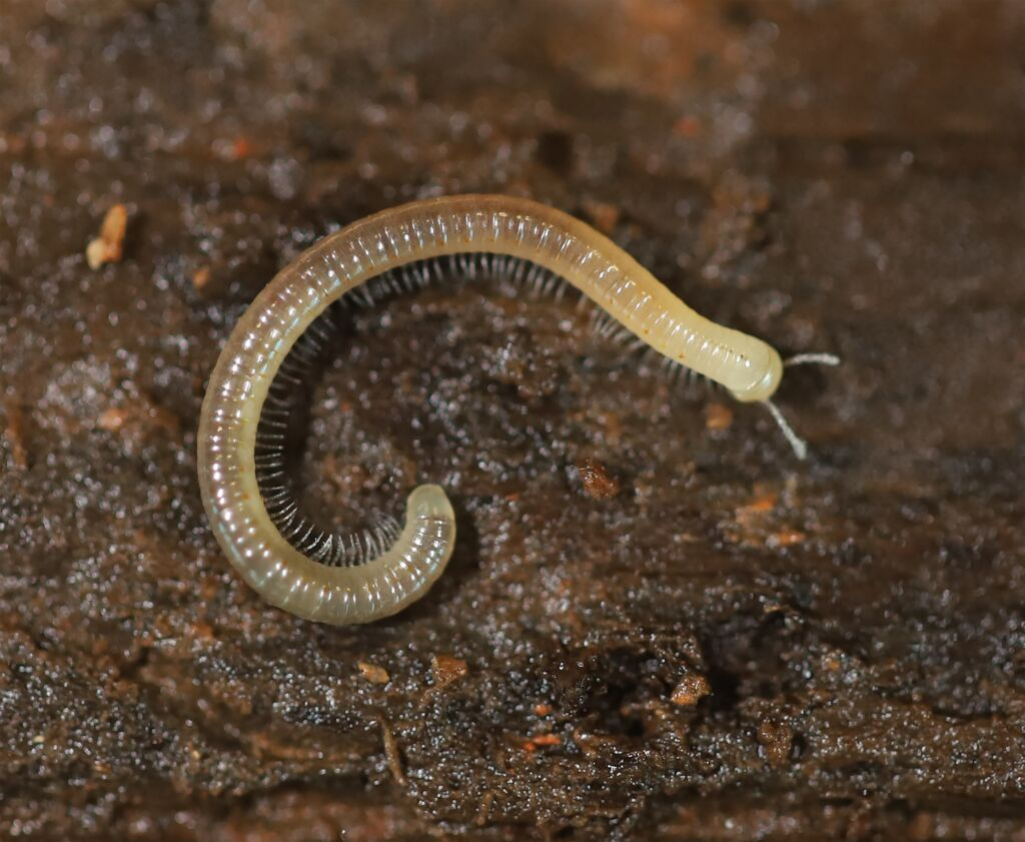
*Cylindroiulusvillumi* Reboleira & Enghoff, 2018, live specimen from Algar do Pena Cave, Portugal.

Figure(s) or Photo(s): Fig. 4

Region for assessment: Europe

#### Editor & Reviewers

#### Geographic range

Biogeographic realm: Palearctic

Countries: Portugal

Map of records (Google Earth): Suppl. materials 1, 8

Basis of EOO and AOO: Known habitat extent

Basis (narrative): Both the extent of occurrence (EOO) and area of occupancy (AOO) are 4 km^2^.

Range description: *Cylindroiulusvillumi* occurs in the Algar do Pena Cave, located in the Santo António Plateau, the central sub-unit of the Estremenho karst massif (Reboleira and Enghoff 2018).

#### Extent of occurrence

EOO (km2): 4

Trend: Unknown

Causes ceased?: Unknown

Causes understood?: Unknown

Causes reversible?: Unknown

Extreme fluctuations?: Unknown

#### Area of occupancy

Trend: Unknown

Causes ceased?: Unknown

Causes understood?: Unknown

Causes reversible?: Unknown

Extreme fluctuations?: Unknown

AOO (km2): 4

#### Locations

Number of locations: 1

Justification for number of locations: *Cylindroiulusvillumi* occurs in a single cave (Reboleira and Enghoff 2018).

Trend: Stable

Justification for trend: This species only occurs in Algar do Pena Cave; therefore, the trend in number of locations is stable.

Extreme fluctuations?: Unknown

#### Population

Number of individuals: Unknown

Trend: Unknown

Causes ceased?: Unknown

Causes understood?: Unknown

Causes reversible?: Unknown

Extreme fluctuations?: Unknown

Population Information (Narrative): Eleven specimens have been collected from the type locality (Reboleira and Enghoff 2018), all having been found in rotten wood pieces under the entrance pit, while its presence is not registered for the rest of the cave, despite intensive monitoring.

#### Subpopulations

Trend: Unknown

Extreme fluctuations?: Unknown

Severe fragmentation?: Unknown

#### Habitat

System: Terrestrial

Habitat specialist: Yes

Habitat (narrative): This species was collected from the largest underground chamber of Portugal, where relative humidity close is up to saturation and temperature is very constant at 13ºC (with a variation of ± 1ºC). Specimens were collected inside a piece of decaying wood, at the base of the entrance pit of the cave, 33 m depth (Reboleira and Enghoff 2018).

Trend in extent, area or quality?: Unknown

##### Habitat

Habitat importance: Major Importance

Habitats: 7.1. Caves and Subterranean Habitats (non-aquatic) - Caves

#### Ecology

Size: 11.4 mm (♂), 13 mm (♀) (body length); 0.7 mm (♂), 0.9 mm (♀) (vertical body diameter).

Generation length (yr): 1

Dependency of single sp?: Unknown

Ecology and traits (narrative): *Cylindroiulusvillumi* is a small to medium-sized blind and depigmented millipede species. Both the holotype and a juvenile male paratype had fungi present on the head and antenna (Reboleira and Enghoff 2018). This species shares habitat with the the springtail *Onychiurusconfugiens* Gama, 1962, the dipluran *Podocampacf.fragiloides* Silvestri, 1932, the spider *Domitiuslusitanicus* (Fage, 1931), the terrestrial isopod *Trichoniscoidesmeridionalis* Vandel, 1946 and the ground beetle *Trechusgamae* Reboleira & Serrano, 2009 ([Bibr B9881898][Bibr B9881907][Bibr B7887211]).

#### Threats

Justification for threats: Algar do Pena is located 300 m from a quarry, where intense quarry activity is currently ongoing, being a source of residues' infiltration and uncontrolled dust release.

##### Threats

Threat type: Ongoing

Threats: 3.2. Energy production & mining - Mining & quarrying6.1. Human intrusions & disturbance - Recreational activities9.1. Pollution - Domestic & urban waste water

#### Conservation

Justification for conservation actions: The habitat of this single cave endemism is inside the Serra de Aire e Candeeiros; however, the species lacks protection status. Measures need to be taken in order to prevent the infiltration of residues from the nearby quarries into the cave. The cave hosts a laboratory for research and has a stainless steel platform that allows touristic visits. Measures to prevent contamination of the cave are already in place (Popova 2022). More research is needed on the life cycle of this species, to estimate total population size and to better comprehend its potential distribution.

##### Conservation actions

Conservation action type: Needed

Conservation actions: 1.1. Land/water protection - Site/area protection2.1. Land/water management - Site/area management2.3. Land/water management - Habitat & natural process restoration4. Education & awareness5.1.3. Law & policy - Legislation - Sub-national level

### Acipes machadoi

#### Species information

Scientific name: Acipesmachadoi

Species authority: Enghoff & Reboleira, 2013

Kingdom: Animalia

Phylum: Arthropoda

Class: Diplopoda

Order: Julida

Family: Blaniulidae

Taxonomic notes: Distinguishable from the other two known blind species, *Acipesandalusius* Enghoff & Mauriès, 1999 and *Acipesbifilum* Enghoff & Reboleira, 2013, by its larger body, less modified first pair of legs in males and by its smooth, rounded apical flange and a very long filamentous tip of the posterior gonopod (Enghoff and Reboleira 2013b).

Region for assessment: Europe

#### Editor & Reviewers

#### Geographic range

Biogeographic realm: Palearctic

Countries: Portugal

Map of records (Google Earth): Suppl. materials 1, 9

Basis of EOO and AOO: Known habitat extent

Basis (narrative): Both the extent of occurrence (EOO) and area of occupancy (AOO) are 4 km^2^.

Max Elevation/Depth (m): 239

Range description: *Acipesmachadoi* occurs in Vale Telheiro Cave, in the Algarve karst massif, southern Portugal (Enghoff and Reboleira 2013b).

#### Extent of occurrence

EOO (km2): 4

Trend: Unknown

Causes ceased?: Unknown

Causes understood?: Unknown

Causes reversible?: Unknown

Extreme fluctuations?: Unknown

#### Area of occupancy

Trend: Unknown

Causes ceased?: Unknown

Causes understood?: Unknown

Causes reversible?: Unknown

Extreme fluctuations?: Unknown

AOO (km2): 4

#### Locations

Number of locations: 1

Justification for number of locations: *Acipesmachadoi* occurs in one location (Enghoff and Reboleira 2013b).

Trend: Stable

Justification for trend: Vale Telheiro Cave is the only known location for this species. Therefore, the trend in number of locations is stable.

Extreme fluctuations?: Unknown

#### Population

Number of individuals: Unknown

Trend: Unknown

Causes ceased?: Unknown

Causes understood?: Unknown

Causes reversible?: Unknown

Extreme fluctuations?: Unknown

Population Information (Narrative): Only one specimen has been collected from the type locality (Enghoff and Reboleira 2013b).

#### Subpopulations

Trend: Unknown

Extreme fluctuations?: Unknown

Severe fragmentation?: Unknown

#### Habitat

System: Terrestrial

Habitat specialist: Yes

Habitat (narrative): *Acipesmachadoi* was collected on the walls of the cave, in the area where roots were hanging from the ceiling. In this cave, humidity levels reach 100% and temperature varies 1ºC throughout the year, with an average of 17.4ºC (Enghoff and Reboleira 2013b).

Trend in extent, area or quality?: Unknown

##### Habitat

Habitat importance: Major Importance

Habitats: 7.1. Caves and Subterranean Habitats (non-aquatic) - Caves

#### Ecology

Size: 24 mm (body length), 0.98 mm (vertical mid-body diameter).

Generation length (yr): 1

Dependency of single sp?: Unknown

Ecology and traits (narrative): *Acipesmachadoi* is a blind and depigmented troglobiont millipede and it is a detritivore species, occupying a basal position in the cave's trophic chain (Enghoff and Reboleira 2013b).

#### Threats

Justification for threats: Vale Telheiro is located 290 m from the closest house and 745 m from the closest urbanisation. The immediate surface of the cave has recently been subject to landfills and the closest road has been enlarged and tarred, facilitating the access to the cave area.

##### Threats

Threat type: Ongoing

Threats: 1.1. Residential & commercial development - Housing & urban areas4. Transportation & service corridors9.1. Pollution - Domestic & urban waste water

#### Conservation

Justification for conservation actions: As the terrain where the cave is located was acquired by the Loulé Town Hall in order to establish a protected area, it is possible to foresee more conservation efforts in the near future. The surface area should be maintained with its natural vegetation cover and cave visitation should be limited. More knowledge on the biology of this species is needed. This species inhabits the richest cave for troglobiont species in Portugal, protecting this species implies the protection this important habitat.

##### Conservation actions

Conservation action type: Needed

Conservation actions: 1.1. Land/water protection - Site/area protection2.1. Land/water management - Site/area management2.3. Land/water management - Habitat & natural process restoration4. Education & awareness5.1.3. Law & policy - Legislation - Sub-national level

### Acipes bifilum

#### Species information

Scientific name: Acipesbifilum

Species authority: Enghoff & Reboleira, 2013

Kingdom: Animalia

Phylum: Arthropoda

Class: Diplopoda

Order: Julida

Family: Blaniulidae

Taxonomic notes: Distinguishable from the other two known blind *Acipes* by the shape of the posterior gonopod which is “curved in the sagittal plane, apically divided into two thread-like structures, one of which corresponds to the apical flange, the other to the filamentous tip” (Enghoff and Reboleira 2013b).

Region for assessment: Europe

#### Editor & Reviewers

#### Geographic range

Biogeographic realm: Palearctic

Countries: Portugal

Map of records (Google Earth): Suppl. materials 1, 10

Basis of EOO and AOO: Known habitat extent

Basis (narrative): Both the extent of occurrence (EOO) and area of occupancy (AOO) are 4 km^2^.

Max Elevation/Depth (m): 85

Range description: *Acipesbifilum* occurs in Senhora Cave, in the Algarve karst massif (Enghoff and Reboleira 2013b).

#### Extent of occurrence

EOO (km2): 4

Trend: Unknown

Causes ceased?: Unknown

Causes understood?: Unknown

Causes reversible?: Unknown

Extreme fluctuations?: Unknown

#### Area of occupancy

Trend: Unknown

Causes ceased?: Unknown

Causes understood?: Unknown

Causes reversible?: Unknown

Extreme fluctuations?: Unknown

AOO (km2): 4

#### Locations

Number of locations: 1

Justification for number of locations: *Acipesbifilum* is known from one single cave location (Enghoff and Reboleira 2013b).

Trend: Stable

Justification for trend: Senhora Cave is the only known location for this species. Therefore, the trend in number of locations is stable.

Extreme fluctuations?: Unknown

#### Population

Number of individuals: Unknown

Trend: Unknown

Causes ceased?: Unknown

Causes understood?: Unknown

Causes reversible?: Unknown

Extreme fluctuations?: Unknown

Population Information (Narrative): Two specimens have been collected from the type locality, despite recurrent sampling efforts, no further specimens have been observed since its description.

#### Subpopulations

Trend: Unknown

Extreme fluctuations?: Unknown

Severe fragmentation?: Unknown

#### Habitat

System: Terrestrial

Habitat specialist: Yes

Habitat (narrative): Senhora Cave is located in the Cerro da Cabeça Mountain in Moncarapacho, Algarve, the southernmost province of Portugal. *Acipesbifilum* was collected on the cave soil, where humidity levels range between 98 and 100% and has an average temperature of 17.7ºC (Enghoff and Reboleira 2013b).

Trend in extent, area or quality?: Unknown

##### Habitat

Habitat importance: Major Importance

Habitats: 7.1. Caves and Subterranean Habitats (non-aquatic) - Caves

#### Ecology

Size: 15 mm (body length), 0.57 mm (vertical mid-body diameter).

Generation length (yr): 1

Dependency of single sp?: Unknown

Ecology and traits (narrative): *Acipesbifilum* is a blind and depigmented troglobiont millipede (Enghoff and Reboleira 2013b). Its type locality, Senhora Cave, is considerably rich in cave-adapted fauna in the national context, harbouring 14 troglobiont species (Enghoff and Reboleira 2013b). The specimens were collected on the cave soil, where humidity levels range between 98 and 100% and has an average temperature of 17.7ºC (Enghoff and Reboleira 2013b).

#### Threats

Justification for threats: Senhora Cave is located 168 m from the closest house and 900 m from an intensive agricultural complex, where strawberries and raspberries are mass-produced. It was subject to profound changes in a failed attempt to transform it into a show cave in the past century, metal and concrete are now in the vertical opening of the cave. This entrance is also used to dump garbage.

##### Threats

Threat type: Ongoing

Threats: 1.1. Residential & commercial development - Housing & urban areas2.2. Agriculture & aquaculture - Wood & pulp plantations4. Transportation & service corridors9.1. Pollution - Domestic & urban waste water

#### Conservation

Justification for conservation actions: The only locality known for this species, the Senhora Cave, is not protected and this single cave endemic millipede lacks protection status.

##### Conservation actions

Conservation action type: Needed

Conservation actions: 1.1. Land/water protection - Site/area protection2.1. Land/water management - Site/area management2.3. Land/water management - Habitat & natural process restoration4. Education & awareness5.1.3. Law & policy - Legislation - Sub-national level

## Discussion

Eight troglobiont species (orders Chordeumatida, Polydesmida and Julida) and one troglophile species (order Callipodida) are known from Portugal. Six troglobiont and one troglophile species occur on the Portuguese mainland and two troglobiont on the Atlantic Island of Madeira. There are no records of cave-dwelling millipede species on the Azores Archipelago (Reboleira and Enghoff 2014a, Reboleira and Enghoff 2017).

Cave-adapted millipedes from Portugal are confined to very limited geographical distributions and ecological features of their subterranean compartments, such as high humidity and lack of light. These troglobiont millipedes ocuppy a basal position in the trophic chains of caves, they are detritivores, which means they feed on decaying organic matter like leaf litter, plant debris and decaying animal remains and excrement (Reboleira and Enghoff 2014a). Their diet plays a vital role in subterranean ecosystem by breaking down organic material and recycling nutrients, contributing to the overall health of the cave ecosystem and underground ecological balance (Reboleira and Enghoff 2014a, Hose et al. 2023).

The order Julida is the most well represented, with three *Cylindroiulus* species (Julidae), one from the Estremenho karst massif (central continental Portugal), two from Madeira Island and two *Acipes* species (Blaniulidae) from the Algarve karst massif (southern continental Portugal) (Enghoff and Reboleira 2013b,Reboleira and Enghoff 2014b, Reboleira and Enghoff 2017, Reboleira and Enghoff 2018). The order Chordeumatida is represented by one monospecific genus *Sireuma*, known only from a single cave in Alentejo and by the smallest species of the genus *Scutogona* in the karst area of Sicó (Enghoff and Reboleira 2013a). The order Polydesmida is represented by one species of the genus *Boreviulisoma* (Paradoxosomatidae), while other taxa remain to be described (Reboleira and Enghoff 2013, Reboleira and Enghoff 2014a).

The callipodid *Lusitanipusalternans* is endemic to central Portugal and, despite being a troglophile species, has only been sampled in caves and in the mesovoid shallow substratum, where it establishes stable populations and has its complete life cycle (Reboleira and Enghoff 2015, Reboleira and Enghoff 2016). This species is also the only known host of the Laboulbeniales fungus *Diplopodomycesveneris* Santam., Enghoff & Reboleira, 2014 (Santamaria et al. 2017), being of particular interest for conservation biology.

Except *Scutogonaminor*, which is found in several caves in the Sicó karst massif, all other troglobiont species of millipedes from continental Portugal are single cave endemics, which entails a great challenge for conservation (Reboleira and Enghoff 2017). The two troglobiont *Cylindroiulus* from Madeira Island are single cave and single island endemics (Reboleira and Enghoff 2014b), which places them on the brink of extinction.

All cave-adapted millipedes from Portugal face ongoing threats related to land-use at surface, pollution infiltration, cave disturbance by visitation and mining and climate change. As for other troglobiont species in Portugal, there is an urgent need to improve the knowledge about population sizes and spacio-temporal dynamics and to understand the limits of their subterranean distribution (Reboleira et al. 2011b, Borges et al. 2019). Moreover, it is important to understand their life cycle, functional ecology and to evaluate their sensitivity to disturbance, in order to implement effective conservation measures (Reboleira and Eusébio 2021, Reboleira et al. 2022).

Protection measures for cave-adapted millipedes in Portugal should include the delimitation of safety areas for conservation perimeters at the surface to avoid infiltration of contaminants, the preservation of natural vegetation at surface to ensure proper nutrient flow towards the underground and to limit the visitation to the caves (Gillieson et al. 2022). Focus should also target raising awareness about the importance of cave ecosystems and implementing measures to preserve cave millipedes and, by extension, subterranean habitats. This includes restricting access and human activities at the surface to certain sensitive cave areas, conducting scientific research to better understand the species biology and ecology and promoting responsible cave tourism practices. Failing to preserve their habitats will inevitably lead to the extinction of cave-adapted millipedes from Portugal.

Discussion

## Supplementary Material

C8F6788B-232E-5718-8873-74DAAF32EE07 10.3897/BDJ.11.e110382.suppl1 Supplementary material 1 Distribution of cave-adapted millipedes in Portugal. Data type Species distribution map Brief description (A) Distribution of cave-adapted millipedes in continental Portugal; (B) Distribution of cave-adapted millipedes in Madeira Island, Portugal.
Species: *Lusitanipusalternans* (pink circle), *Sireumanobile* (green diamond), *Scutogonaminor* (blue cross), *Boreviulisomabarrocalense* (yellow triangle), *Cylindroiulusjulesvernei* (light purple hexagon), *Cylindroiulusoromii* (dark purple hexagon), *Cylindroiulusvillumi* (blue hexagon), *Acipesmachadoi* (red square), *Acipesbifilum* (orange square). File: oo_654729.tif https://binary.pensoft.net/file/654729 A.S.P.S. Reboleira, R.P. Eusébio

34F049E3-FCC7-5628-90D8-BBC2D862840E 10.3897/BDJ.11.e110382.suppl2 Supplementary material 2 Distribution of the millipede *Lusitanipusalternans*. Data type Species distribution map Brief description *Lusitanipusalternans* distribution: d'el Rey, Arrifana, Cerâmica, Soprador do Carvalho, Corujeiras, Fonte Grande and Buraco da Moura caves and Vale do Poio mesovoid shallow substratum. File: oo_677404.png https://binary.pensoft.net/file/677404 A.S.P.S. Reboleira, R.P. Eusébio

AA8E2A68-BD3D-52BA-BAC7-A9795F582545 10.3897/BDJ.11.e110382.suppl3 Supplementary material 3 Distribution of the millipede *Sireumanobile*. Data type Species distribution map Brief description *Sireumanobile* distribution: Algar de Santo António, Estremoz-Cano karst massif. File: oo_655202.png https://binary.pensoft.net/file/655202 A.S.P.S. Reboleira, R.P. Eusébio

7BC8E37D-CAE3-5B4D-8946-9D445EE80447 10.3897/BDJ.11.e110382.suppl4 Supplementary material 4 Distribution of the millipede *Scutogonaminor*. Data type Species distribution map Brief description *Scutogonaminor* distribution: Santa Maria da Estrela, Cerâmica and Arrifana caves, Sicó karst massif. File: oo_655264.png https://binary.pensoft.net/file/655264 A.S.P.S. Reboleira, R.P. Eusébio

7577623D-125F-5DE1-A220-B226B0DAA54F 10.3897/BDJ.11.e110382.suppl5 Supplementary material 5 Distribution of the millipede *Boreviulisomabarrocalense*. Data type Species distribution map Brief description *Boreviulisomabarrocalense* distribution: Vale Telheiro Cave, Algarve karst massif. File: oo_658648.png https://binary.pensoft.net/file/658648 A.S.P.S. Reboleira, R.P. Eusébio

926782B7-DD5B-54DC-B0FB-0CB8ED193F1C 10.3897/BDJ.11.e110382.suppl6 Supplementary material 6 Distribution of the millipede *Cylindroiulusjulesvernei*. Data type Species distribution map Brief description *Cylindroiulusjulesverne* distribution: São Vicente Cave, Madeira Archipelago. File: oo_658656.png https://binary.pensoft.net/file/658656 A.S.P.S. Reboleira, R.P.Eusébio

65F614ED-FC00-57EA-8D50-1F22D3138737 10.3897/BDJ.11.e110382.suppl7 Supplementary material 7 Distribution of the millipede *Cylindroiulusoromii*. Data type Species distribution map Brief description *Cylindroiulusoromii* distribution: Landeiros Cave, Madeira Archipelago. File: oo_658878.png https://binary.pensoft.net/file/658878 A.S.P.S. Reboleira, R.P. Eusébio

DBE45FE5-659E-5C7A-AF2F-5D6FC7A1E27C 10.3897/BDJ.11.e110382.suppl8 Supplementary material 8 Distribution of the millipede *Cylindroiulusvillumi*. Data type Species distribution map Brief description *Cylindroiulusvillumi* distribution: Algar do Pena Cave, Estremenho karst massif. File: oo_659034.png https://binary.pensoft.net/file/659034 A.S.P.S. Reboleira, R.P. Eusébio

A7381A27-9714-5048-BAB8-D341FE79C8C0 10.3897/BDJ.11.e110382.suppl9 Supplementary material 9 Distribution of the millipede *Acipesmachadoi*. Data type Species distribution map Brief description *Acipesmachadoi* distribution: Vale Telheiro Cave, Algarve karst massif. File: oo_659130.png https://binary.pensoft.net/file/659130 A.S.P.S. Reboleira, R.P. Eusébio

310FE7A2-15A0-54D8-9C61-E91BE46DFD9D 10.3897/BDJ.11.e110382.suppl10 Supplementary material 10 Distribution of the millipede *Acipesbifilum*. Data type Species distribution map Brief description *Acipesbifilum* distribution: Senhora Cave, Algarve karst massif. File: oo_659149.png https://binary.pensoft.net/file/659149 A.S.P.S. Reboleira & R.P. Eusébio

## References

[B9881877] Borges Paulo, Lamelas-Lopez Lucas, Amorim Isabel, Danielczak Anja, Boieiro Mário, Rego Carla, Wallon Sophie, Nunes Rui, Cardoso Pedro, Hochkirch Axel (2019). Species conservation profiles of cave-dwelling arthropods from Azores, Portugal. Biodiversity Data Journal.

[B7729197] Directive H. (1992). Council directive 92/43/EEC of 21 May 1992 on the conservation of natural habitats and of wild fauna and flora.. Official Journal of the European Union.

[B7704915] Enghoff H., Reboleira A. S.P.S. (2013). A new cave-dwelling millipede of the genus *Scutogona* from central Portugal (Diplopoda, Chordeumatida, Chamaesomatidae). Zootaxa.

[B7704951] Enghoff H., Reboleira A. S.P.S. (2013). Subterranean species of *Acipes* Attems, 1937 (Diplopoda, Julida, Blaniulidae). Zootaxa.

[B10478127] Gilgado José Domingo, Martínez-Pillado Virginia, Prieto Carlos E. (2020). A new green-coloured *Lusitanipus* Mauriès, 1978 from the Iberian Peninsula (Diplopoda: Callipodida: Dorypetalidae). European Journal of Taxonomy.

[B9879811] Gillieson David, Gunn John, Auler Augusto, Bolger Terry (2022). Guidelines for cave and karst protection: second edition.

[B10281864] Hose Grant C., Di Lorenzo Tiziana, Fillinger Lucas, Galassi Diana Maria Paola, Griebler Christian, Hahn Hans Juergen, Handley Kim M., Korbel Kathryn, Reboleira Ana Sofia, Siemensmeyer Tobias, Spengler Cornelia, Weaver Louise, Weigand Alexander (2023). Assessing groundwater ecosystem health, status, and services. Groundwater Ecology and Evolution.

[B7729206] ICNB Grutas não exploradas pelo turismo. Plano Sectorial da Rede Natura 2000: Habitats Naturais (8130).. http://www2.icnf.pt/portal/pn/biodiversidade/rn2000/resource/doc/rn-plan-set/hab/hab-8310/view.

[B7778141] ICNF Rede nacional de áreas protegidas (RNAP). https://sig.icnf.pt/portal/home/item.html?id=02b7a03f8fbd4dada77f5f3e5f91f186.

[B10478176] Ilić Bojan, Unković Nikola, Ćirić Ana, Glamočlija Jasmina, Ljaljević Grbić Milica, Raspotnig Günther, Bodner Michaela, Vukojević Jelena, Makarov Slobodan (2019). Phenol-based millipede defence: antimicrobial activity of secretions from the Balkan endemic millipede *Apfelbeckiainsculpta* (L. Koch, 1867) (Diplopoda: Callipodida). The Science of Nature.

[B7712139] Kime R. D., Enghoff H. (2011). Atlas of European millipedes (Class Diplopoda). Vol. 1. Orders Polyxenida, Glomerida, Platydesmida, Siphonocryptidae, Polyzoniida, Callipodida, Polydesmida.

[B7712074] Kime Richard Desmond, Enghoff Henrik (2017). Atlas of European millipedes. Vol. 2. Order Julida (Class Diplopoda). European Journal of Taxonomy.

[B7712104] Kime Richard Desmond, Enghoff Henrik (2021). Atlas of European millipedes. Vol. 3. Order Chordeumatida (Class Diplopoda). European Journal of Taxonomy.

[B7857298] Liu Weixin, Golovatch Sergei, Wesener Thomas, Tian Mingyi (2017). Convergent evolution of unique morphological adaptations to a subterranean environment in cave millipedes (Diplopoda).. PLOS One.

[B9814650] Popova E. (2022). Identification of threats on geodiversity and biodiversity in Pena Cave, Portugal: contributions to improve cave management.

[B8059621] Ravn Nynne Rand, Michelsen Anders, Reboleira Ana Sofia P. S. (2020). Decomposition of organic matter in caves. Frontiers in Ecology and Evolution.

[B7712156] Reboleira Ana, Gonçalves Fernando, Oromí Pedro (2013). Literature survey, bibliographic analysis and a taxonomic catalogue of subterranean fauna from Portugal. Subterranean Biology.

[B9881898] Reboleira Ana Sofia P. S., Gonçalves Fernando J., Serrano Artur R. M. (2009). Two new species of cave dwelling *Trechus* Clairville, 1806 of the *fulvus*-group (Coleoptera, Carabidae, Trechinae) from Portugal. Deutsche Entomologische Zeitschrift.

[B7887229] Reboleira Ana Sofia, Sendra Alberto, Gonçalves F., Oromí Pedro (2010). The first hypogean dipluran from Portugal: description of a new species of the genus *Litocampa* (Diplura: Campodeidae). Zootaxa.

[B7887202] Reboleira A. S.P.S., Zaragoza J. A., Gonçalves F., Oromí P. (2010). *Titanobochica*, surprising discovery of a new cave-dwelling genus from southern Portugal (Arachnida: Pseudoscorpiones: Bochicidae). Zootaxa.

[B9881907] Reboleira A. S.P.S., Ortuño V. M. (2011). Description of the larva and female genitalia of *Trechusgamae* with data on its ecology. Bulletin of Insectology.

[B7785657] Reboleira A. S.P.S., Gonçalves F., Oromí P. (2011). On the Iberian endemic subgenusLathromene Koch (Coleoptera: Staphylinidae: Paederinae): description of the first hypogean *Domene* Fauvel, 1872 from Portugal. Zootaxa.

[B7712165] Reboleira Ana Sofia, Borges Paulo, Gonçalves Fernando, Serrano Artur, Oromí Pedro (2011). The subterranean fauna of a biodiversity hotspot region - Portugal: an overview and its conservation. International Journal of Speleology.

[B7887242] Reboleira A. S.P.S. (2012). Biodiversity and conservation of subterranean fauna of Portuguese karst.

[B7887174] Reboleira A. S.P.S., Gonçalves F., Oromí P., Mendes L. F. (2012). *Squamatiniaalgharbica* gen. n. sp. n., a remarkable new Coletiniinae silverfish (Zygentoma: Nicoletiidae) from caves in southern Portugal. Zootaxa.

[B7712214] Reboleira A. S.P.S., Enghoff H. (2013). The genus *Boreviulisoma* Brolemann, 1928—an Iberian-N African outlier of a mainly tropical tribe of millipedes (Diplopoda: Polydesmida: Paradoxosomatidae). Zootaxa.

[B7712205] Reboleira A. S.P.S., Enghoff Henrik (2014). Millipedes (Diplopoda) from Caves of Portugal. Journal of Cave and Karst Studies.

[B7712251] Reboleira A. S.P.S., Enghoff H. (2014). Insular species swarm goes underground: two new troglobiont *Cylindroiulus* millipedes from Madeira (Diplopoda: Julidae). Zootaxa.

[B7729516] Reboleira A. S.P.S., Enghoff H. (2014). *Sireuma*, a new genus of subterranean millipedes from the Iberian Peninsula (Diplopoda, Chordeumatida, Opisthocheiridae). Zootaxa.

[B7712223] Reboleira A. S.P.S., Enghoff H. (2015). Redescription of *Lusitanipusalternans* (Verhoeff, 1893) (Diplopoda, Callipoda, Dorypetalidae) and ecological data on its *Laboulbeniales* ectoparasites in caves. Zootaxa.

[B7887211] Reboleira Ana Sofia P. S., Gonçalves Fernando, Oromí Pedro, Taiti Stefano (2015). The cavernicolous Oniscidea (Crustacea: Isopoda) of Portugal. European Journal of Taxonomy.

[B7712233] Reboleira Ana Sofia P. S., Enghoff Henrik (2016). Mud and silk in the dark: A new type of millipede moulting chamber and first observations on the maturation moult in the order Callipodida. Arthropod Structure & Development.

[B7712196] Reboleira A. S.P.S., Enghoff H. (2017). Subterranean millipedes (Diplopoda) of the Iberian Peninsula. Zootaxa.

[B7779854] Reboleira Ana Sofia P. S., Fresnada Javier, Salgado José Maria (2017). A new species of *Speonemadus* from Portugal, with the revision of the *escalerai*-group (Coleoptera, Leiodidae). European Journal of Taxonomy.

[B7704942] Reboleira Ana Sofia P. S., Enghoff Henrik (2018). First continental troglobiont *Cylindroiulus* millipede (Diplopoda, Julida, Julidae). ZooKeys.

[B7712147] Reboleira Ana Sofia, Eusébio Rita (2021). Cave-adapted beetles from continental Portugal. Biodiversity Data Journal.

[B7884000] Reboleira Ana Sofia P S, Zaragoza Juan A, Gonçalves Fernando, Oromi Pedro (2013). On hypogean *Roncocreagris* (Arachnida: Pseudoscorpiones: Neobisiidae) from Portugal, with descriptions of three new species.. Zootaxa.

[B7887220] Reboleira Ana Sofia P S, Eusébio Rita P, Taiti Stefano (2022). Species conservation profiles of cave-adapted terrestrial isopods from Portugal.. Biodiversity Data Journal.

[B7729180] Ribera Ignacio, Reboleira Ana Sofia P. S. (2019). The first stygobiont species of Coleoptera from Portugal, with a molecular phylogeny of the *Siettitia* group of genera (Dytiscidae, Hydroporinae, Hydroporini, Siettitiina). ZooKeys.

[B7712242] Santamaria Sergi, Enghoff Henrik, Reboleira Ana Sofia P. S. (2017). Laboulbeniales on millipedes: the genera *Diplopodomyces* and *Troglomyces*. Mycologia.

[B7883991] Sendra A., Reboleira A. S.P.S. (2020). Euro-Mediterranean fauna of Campodeinae (Campodeidae, Diplura). European Journal of Taxonomy.

[B7884009] Zaragoza J. A., Reboleira A. S.P.S (2018). Five new hypogean *Occidenchthonius* (Pseudoscorpiones, Chthoniidae) from Portugal. Journal of Arachnology.

