# Supplementary figures and images for: Cave-adapted millipedes from Portugal: species conservation profiles
Source: Biodivers Data J. 2023 Nov 10;11:e110382. doi: 10.3897/BDJ.11.e110382 (PMC10838078; doi:10.3897/BDJ.11.e110382)

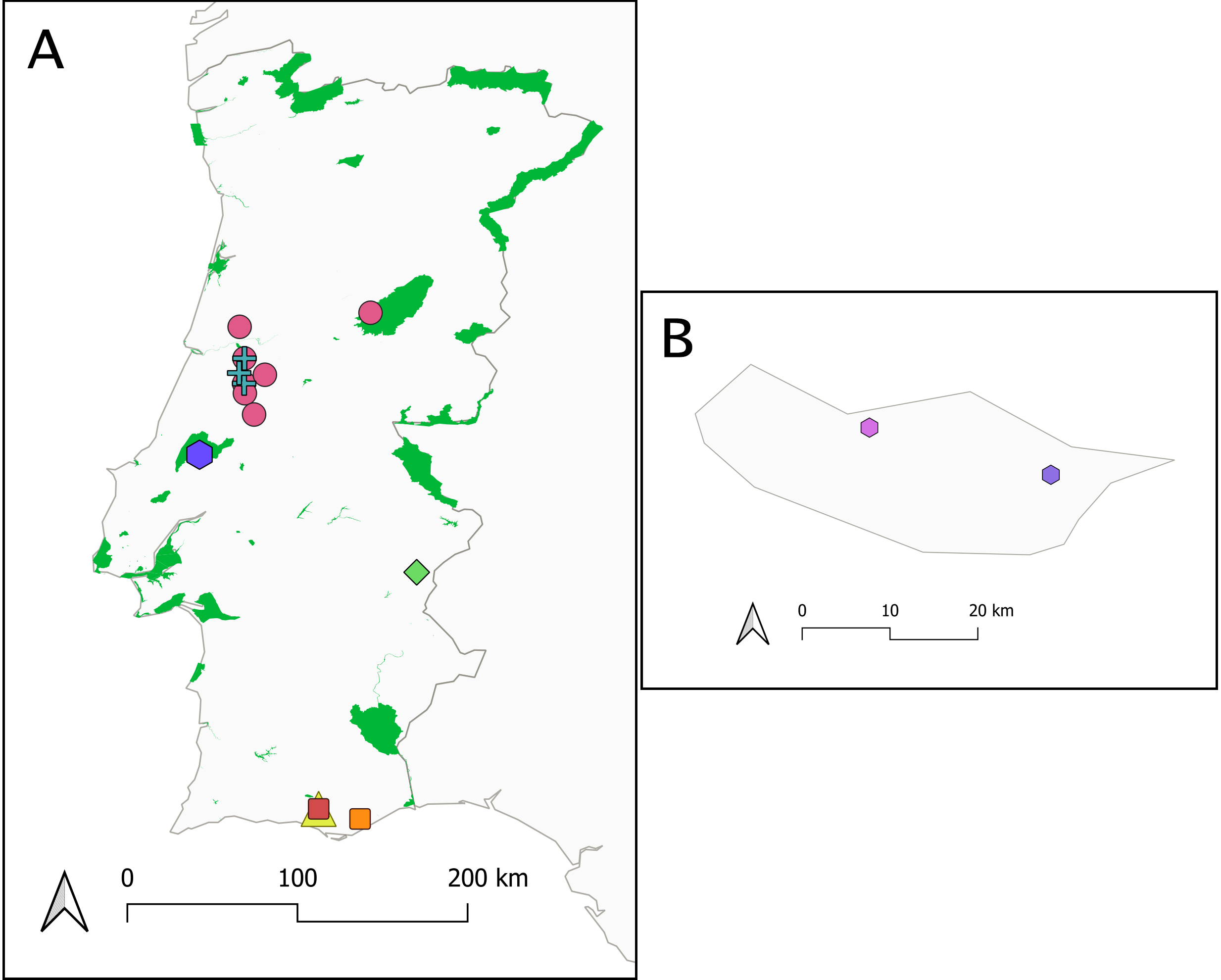

Supplement: Supplementary material 1 — Distribution of cave-adapted millipedes in Portugal. [file bdj-11-e110382-s001.tif]

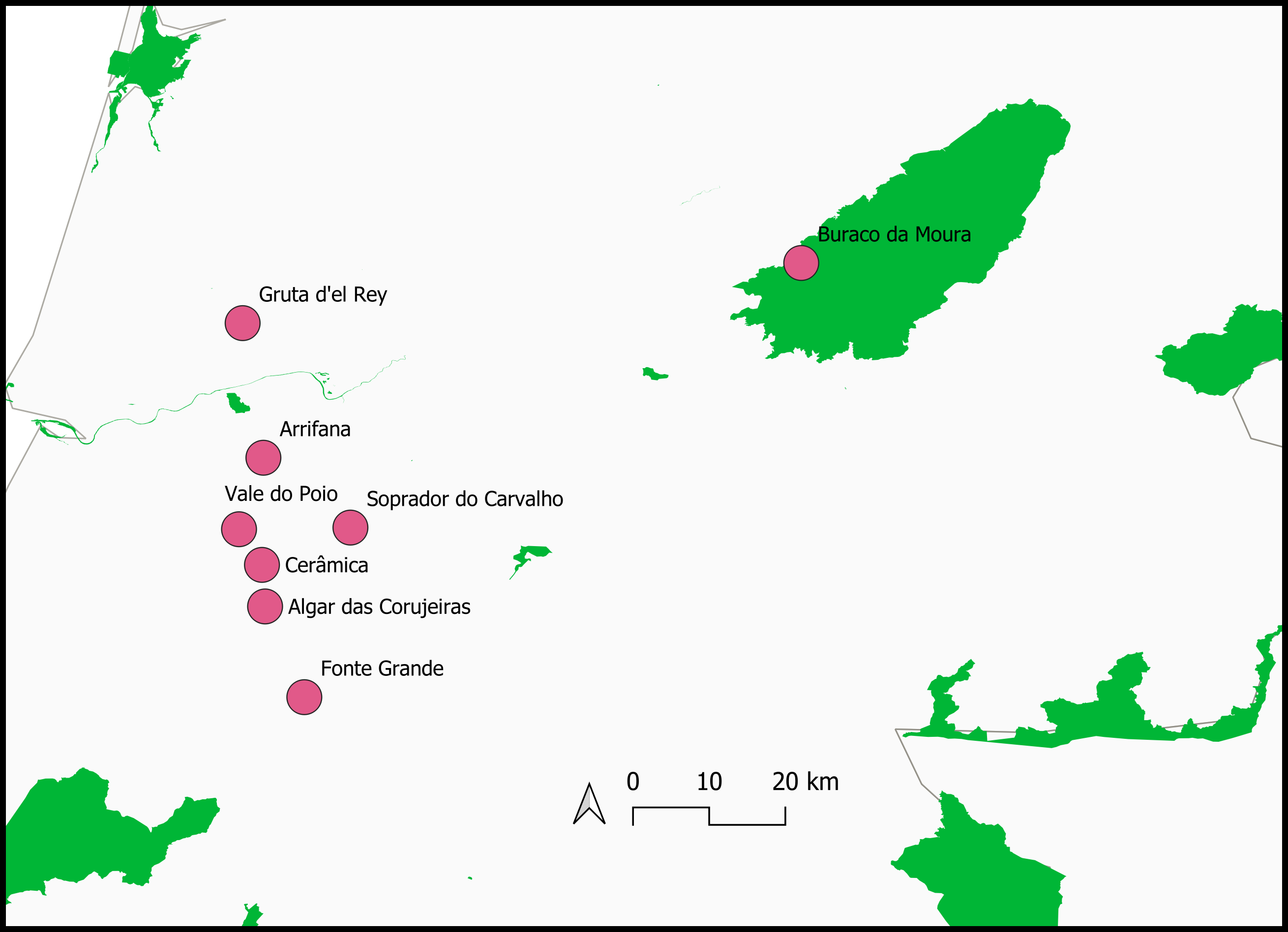

Supplement: Supplementary material 2 — Distribution of the millipede Lusitanipusalternans. [file bdj-11-e110382-s002.png]

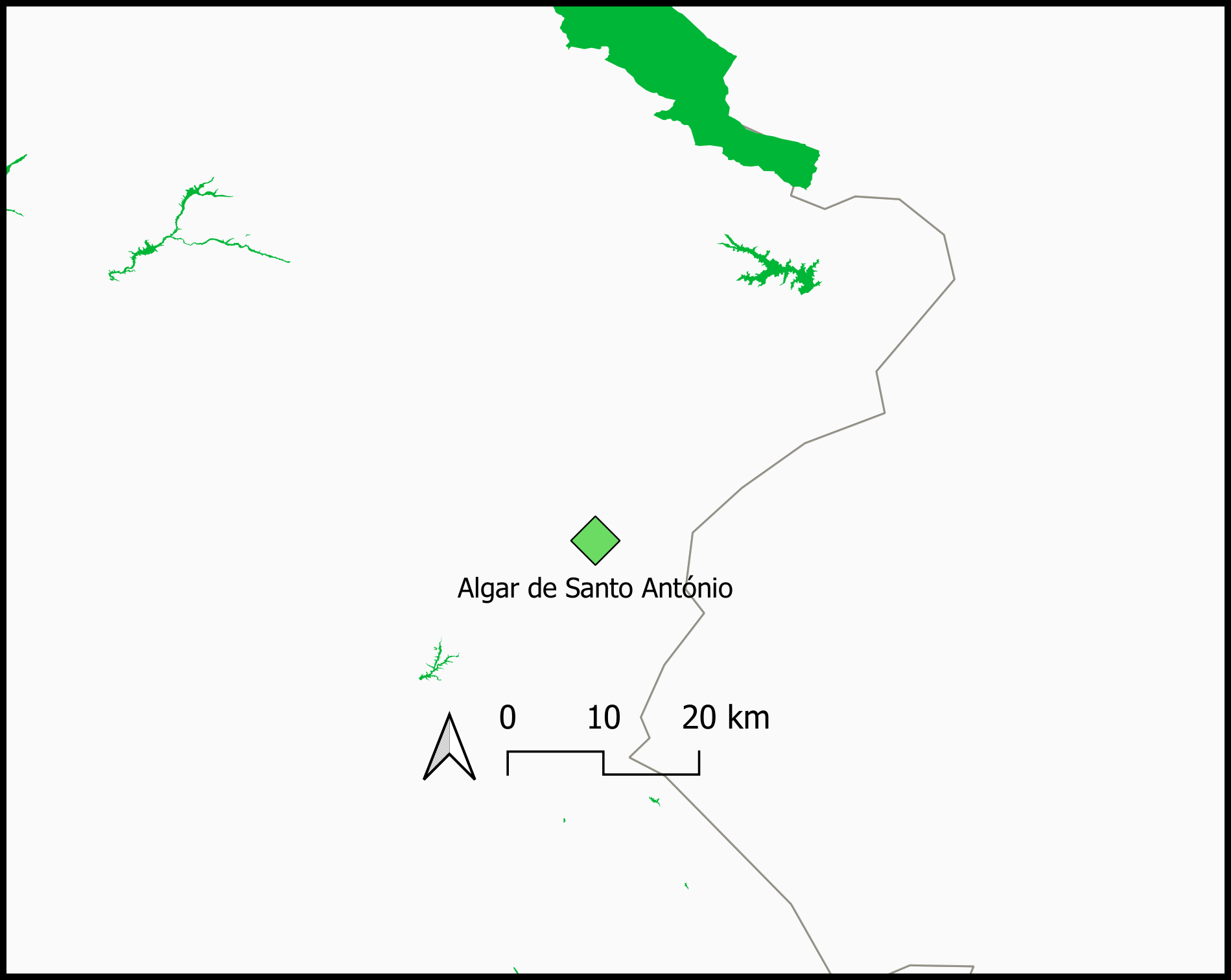

Supplement: Supplementary material 3 — Distribution of the millipede Sireumanobile. [file bdj-11-e110382-s003.png]

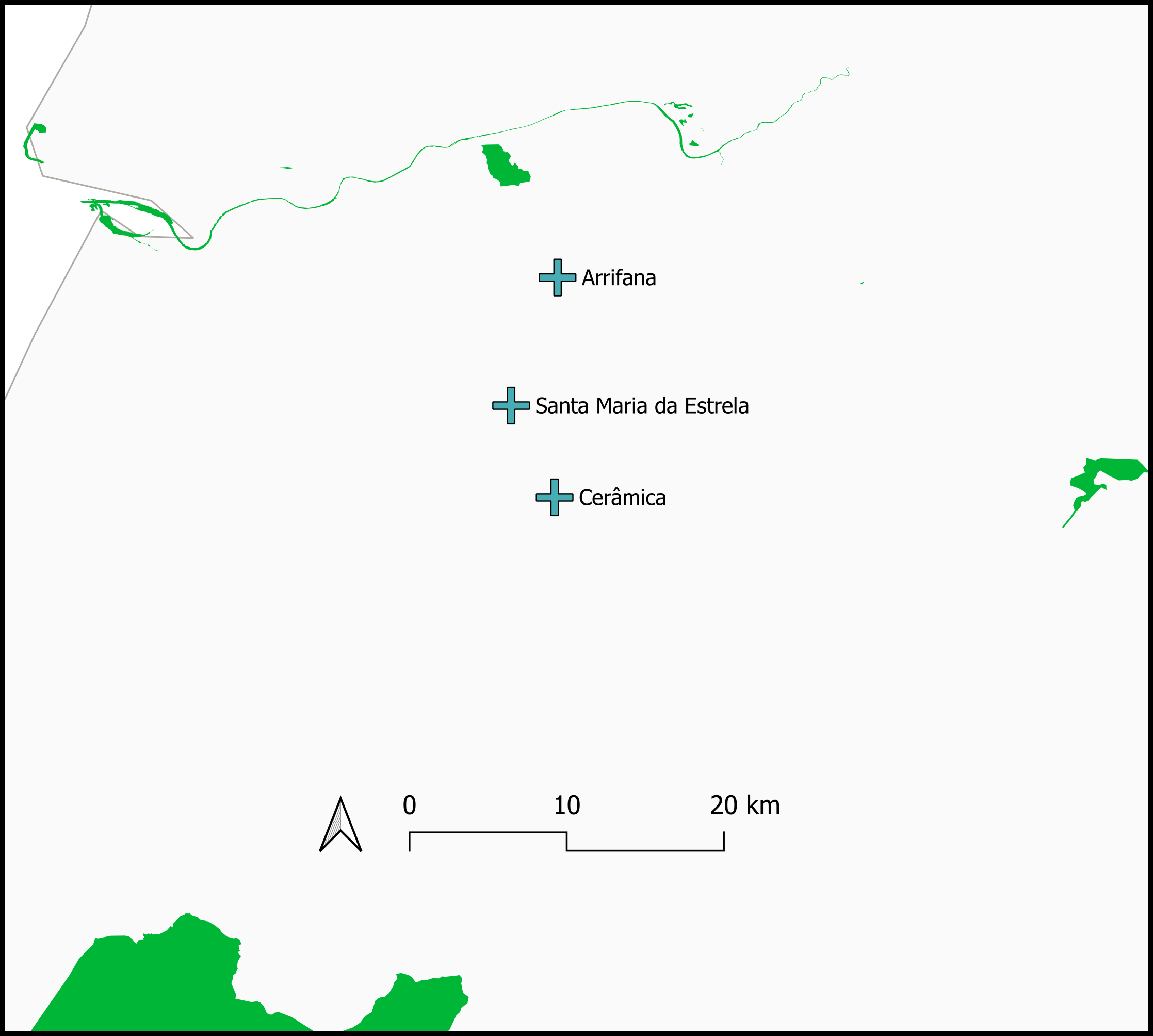

Supplement: Supplementary material 4 — Distribution of the millipede Scutogonaminor. [file bdj-11-e110382-s004.png]

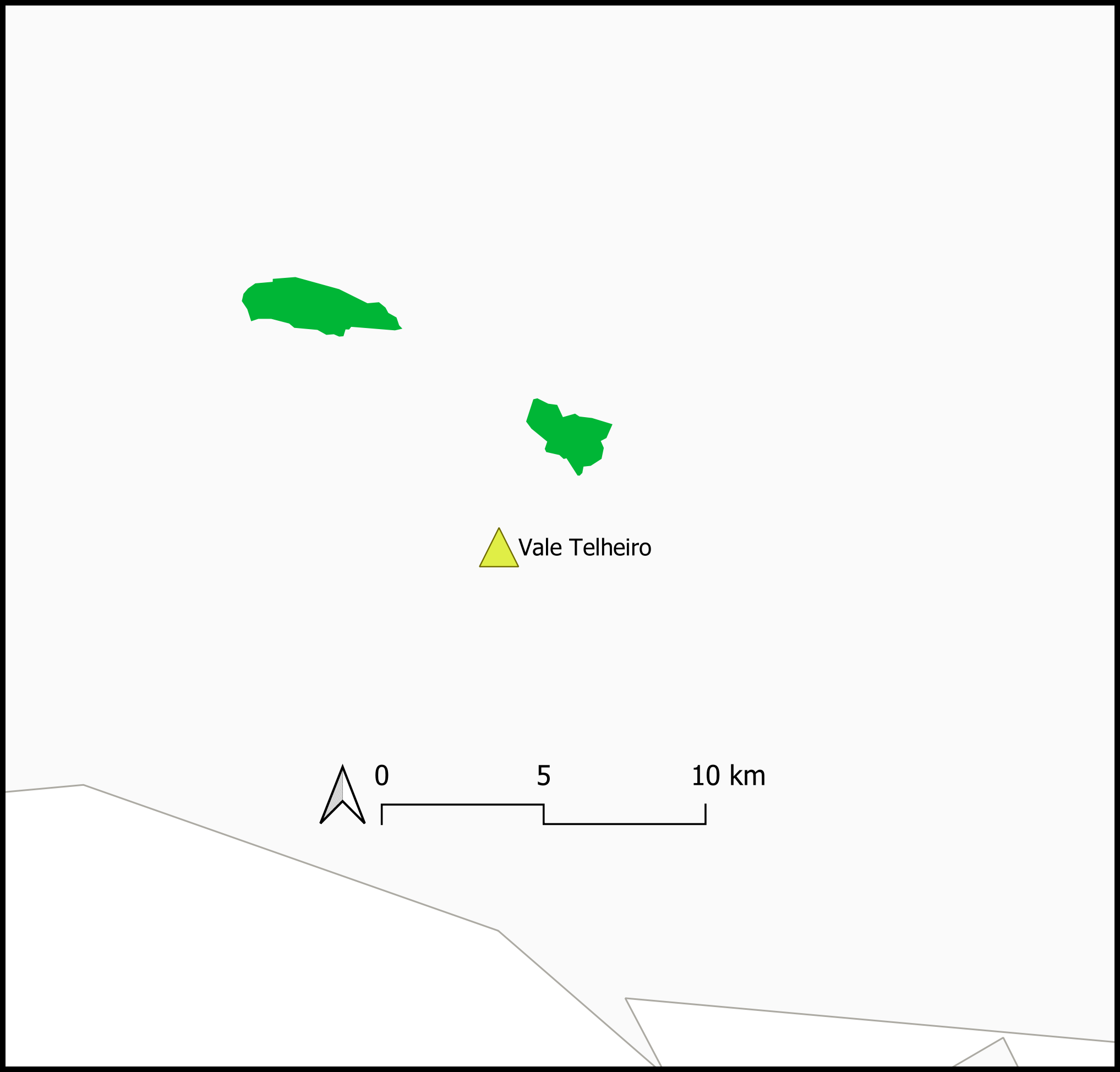

Supplement: Supplementary material 5 — Distribution of the millipede Boreviulisomabarrocalense. [file bdj-11-e110382-s005.png]

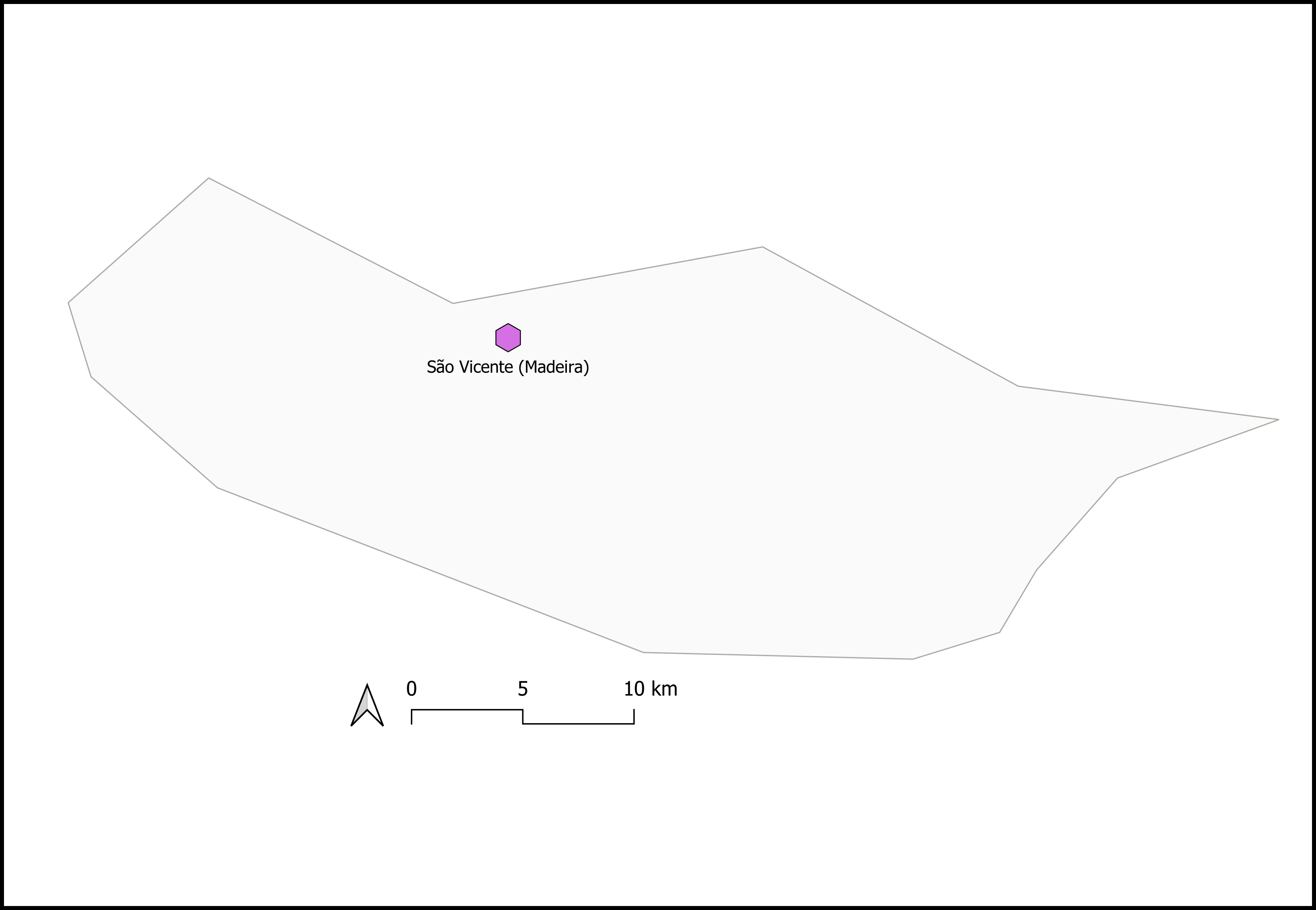

Supplement: Supplementary material 6 — Distribution of the millipede Cylindroiulusjulesvernei. [file bdj-11-e110382-s006.png]

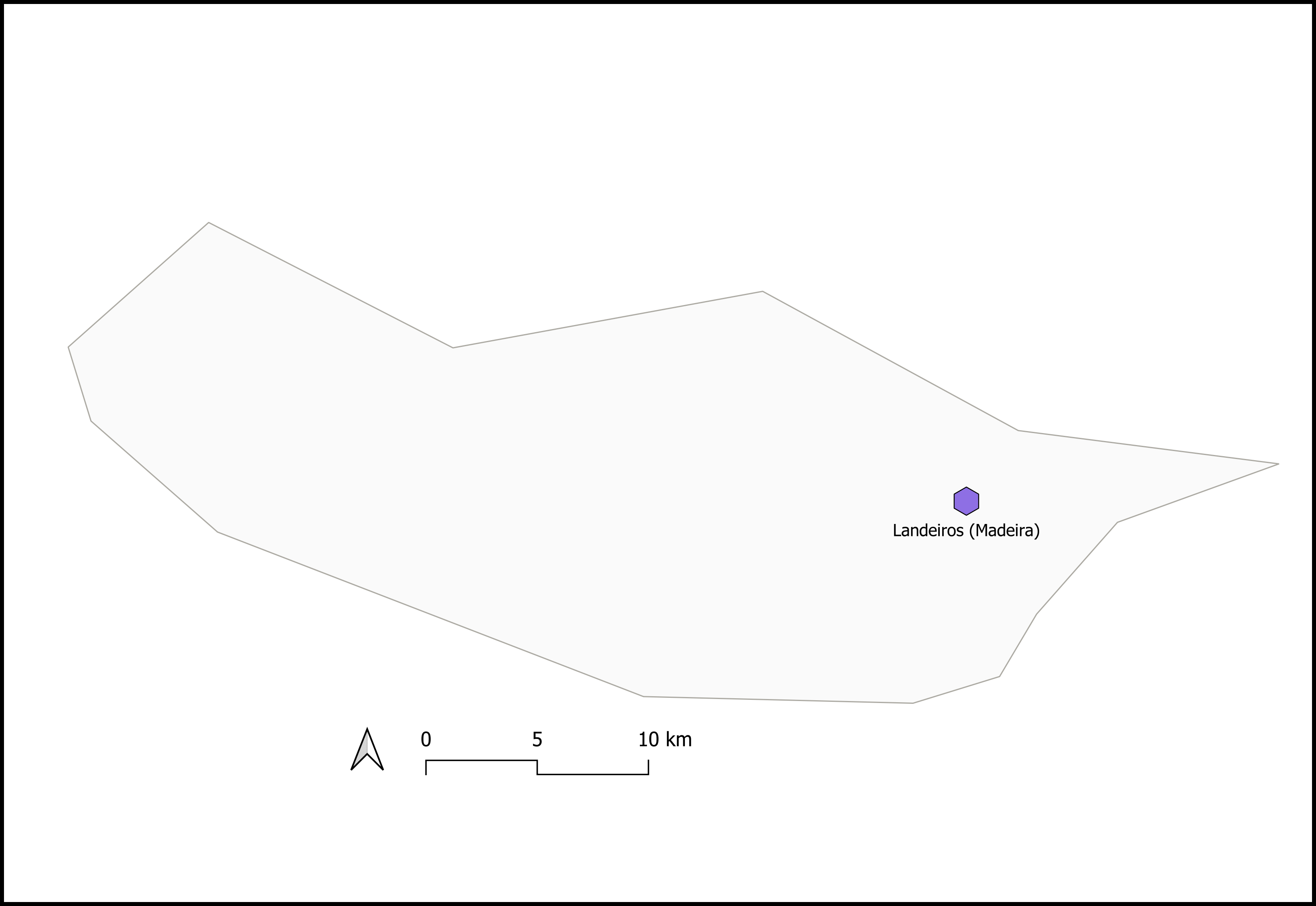

Supplement: Supplementary material 7 — Distribution of the millipede Cylindroiulusoromii. [file bdj-11-e110382-s007.png]

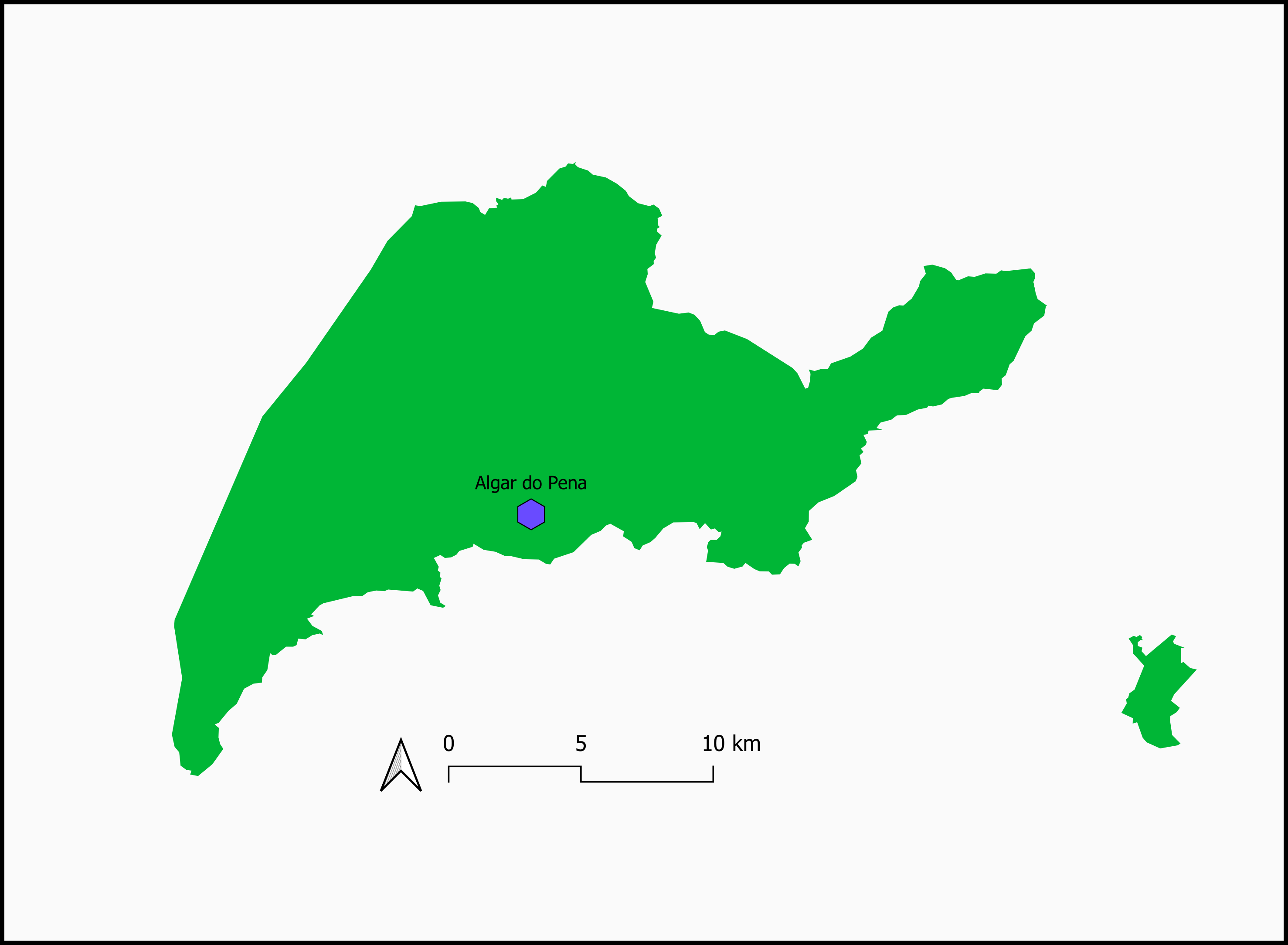

Supplement: Supplementary material 8 — Distribution of the millipede Cylindroiulusvillumi. [file bdj-11-e110382-s008.png]

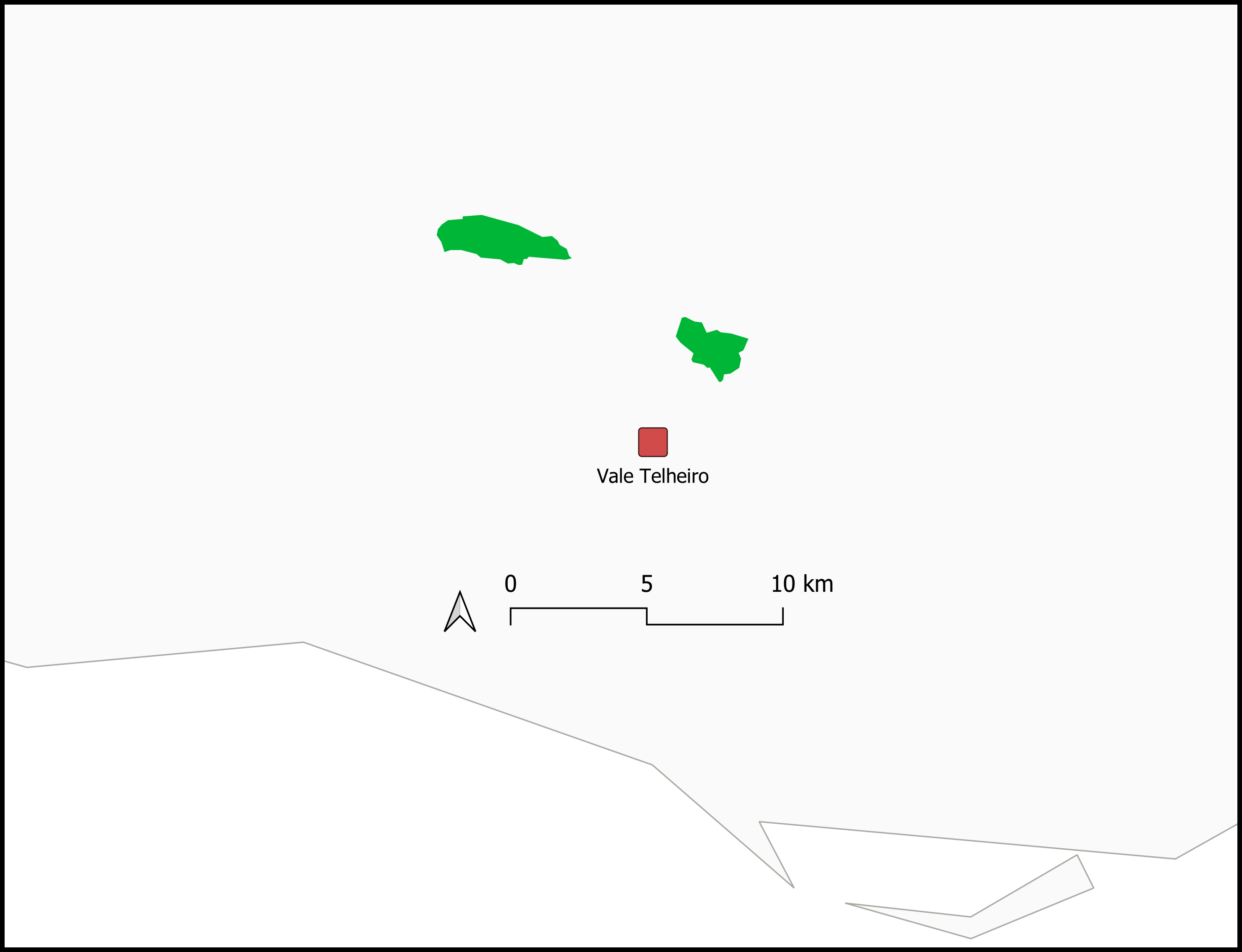

Supplement: Supplementary material 9 — Distribution of the millipede Acipesmachadoi. [file bdj-11-e110382-s009.png]

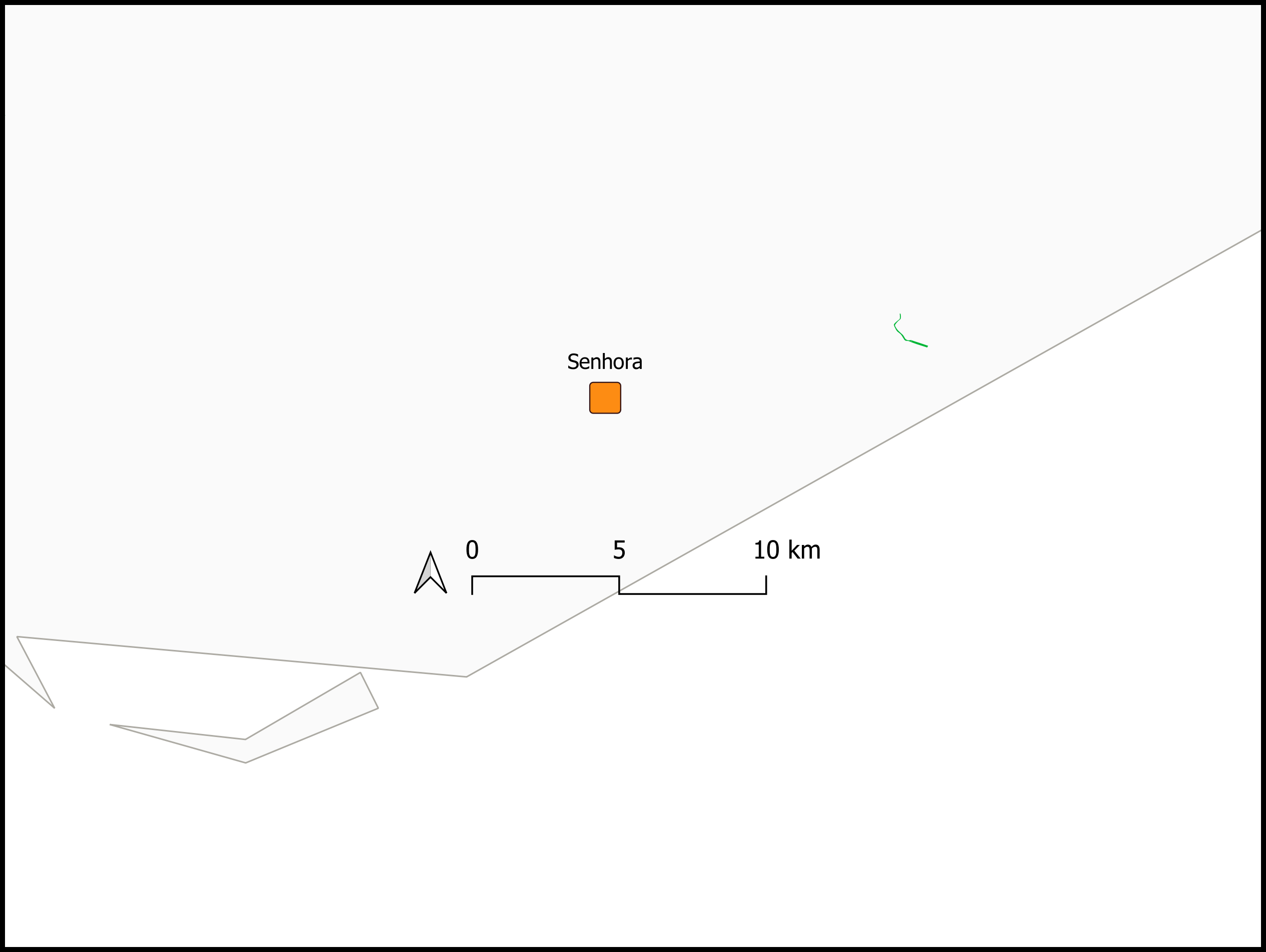

Supplement: Supplementary material 10 — Distribution of the millipede Acipesbifilum. [file bdj-11-e110382-s010.png]
